# Full-Length Transcriptome Assembly of Italian Ryegrass Root Integrated with RNA-Seq to Identify Genes in Response to Plant Cadmium Stress

**DOI:** 10.3390/ijms21031067

**Published:** 2020-02-06

**Authors:** Zhaoyang Hu, Yufei Zhang, Yue He, Qingqing Cao, Ting Zhang, Laiqing Lou, Qingsheng Cai

**Affiliations:** College of Life Sciences, Nanjing Agricultural University, Nanjing 210095, China; 2016216002@njau.edu.cn (Z.H.); 2018116014@njau.edu.cn (Y.Z.); 2018116015@njau.edu.cn (Y.H.); 2016116018@njau.edu.cn (Q.C.); 2016116017@njau.edu.cn (T.Z.); qscai@njau.edu.cn (Q.C.)

**Keywords:** alternative splicing, cadmium, Italian ryegrass root, transcriptome, cadmium, *LmAUX1*

## Abstract

Cadmium (Cd) is a toxic heavy metal element. It is relatively easily absorbed by plants and enters the food chain, resulting in human exposure to Cd. Italian ryegrass (*Lolium multiflorum* Lam.), an important forage cultivated widely in temperate regions worldwide, has the potential to be used in phytoremediation. However, genes regulating Cd translocation and accumulation in this species are not fully understood. Here, we optimized PacBio ISO-seq and integrated it with RNA-seq to construct a de novo full-length transcriptomic database for an un-sequenced autotetraploid species. With the database, we identified 2367 differentially expressed genes (DEGs) and profiled the molecular regulatory pathways of Italian ryegrass with Gene Ontology (GO) and Kyoto Encyclopedia of Genes and Genomes (KEGG) analysis in response to Cd stress. Overexpression of a DEG *LmAUX1* in *Arabidopsis thaliana* significantly enhanced plant Cd concentration. We also unveiled the complexity of alternative splicing (AS) with a genome-free strategy. We reconstructed full-length UniTransModels using the reference transcriptome, and 29.76% of full-length models had more than one isoform. Taken together, the results enhanced our understanding of the genetic diversity and complexity of Italian ryegrass under Cd stress and provided valuable genetic resources for its gene identification and molecular breeding.

## 1. Introduction

The genus *Lolium* L., belonging to the *Poaceae* family, is native to Europe, North Africa, and temperate Asia and has been introduced to almost all temperate regions in the world [1]. Italian ryegrass (*L. multiflorum* Lam.) and perennial ryegrass (*L. perenne* L.) are two important species, and both of them are valuable forage grasses. Because of their desirable characteristics, such as high yields, tolerance to grazing, rapid establishment, and high palatability and digestibility for ruminant animals, they have been cultivated through hybrid or molecular breeding in the last hundred years [2]. Moreover, molecular breeding has played an increasingly important role in breeding programs of late [3]. In many plants, it has shown promise in increasing yield as well as resistance to multiple biotic and abiotic stresses. However, molecular breeding depends on the availability of a reference genome. Until now, in species of the *Lolium* genus, only a draft genome of perennial ryegrass (*L. perenne* L.) was reported [4]. Additionally, there are still gaps in the genome, which limits the understanding of the molecular regulation mechanism, especially for closely related species, such as Italian ryegrass, so it provides little reference value. Italian ryegrass, also known as annual ryegrass, is used for turf, forage, and quick cover in the event of erosion [5], and it also has some new uses, such as lignocellulosic ethanol conversion programs [6] and phytoremediation [7].

Cadmium (Cd) is a widespread heavy metal element in nature, is highly toxic to almost all plants and animals, and is without any known biological functions [8]. Cd uptake by plants from Cd-contaminated soils happens relatively easily, resulting in human Cd exposure through the food chain [9]. Soil Cd pollution has drawn public attention due to its direct adverse impact on plant growth and the uncertainties regarding the safety of produce [10]. Many previous studies have shown that Cd exposure may cause a series of damages to plants [11], such as lipid peroxidation, enzyme inactivation, an overproduction of reactive oxygen species (ROSs), a disturbed assimilation of essential elements (for example, copper, iron, and zinc), activated programmed cell death (PCD), and membrane and DNA damage [12,13]. Additionally, these damages eventually lead to various toxic phenotypes, such as leaf yellowing, growth reduction, plant withering, or even death [14]. In recent years, common plant mechanisms, in response to Cd stress, have been revealed, such as Cd transporters, Cd cellular compartmentation, and chelation [15]. In *Sedum alfredii*, a hyperaccumulator, an apoplastic pathway mediated by abscisic acid (ABA), regulated the uptake of Cd [16]; in rice, several transporters participating in the absorption, transport, and distribution of Cd have been characterized, including OsHMA3 [8], OsNramp5 [17], and OsCAL1 [18]; in *Arabidopsis thaliana*, the ABC-type transporter AtABCC3 induced by Cd increased plant Cd tolerance mediated by phytochelatin [19].

RNA-seq, as a high-throughput next-generation sequencing (NGS) technique, has become an indispensable tool for transcriptome-wide analysis of differential gene expression and gene regulatory networks. It has recently been widely used for researching plants in response to various stresses, including Cd stress, in such plants as switchgrass [20], rice [21], and *S. alfredii* Hance [22]. However, the limitations of RNA-seq, i.e., short read lengths and amplification biases, restrict researchers to accurately obtain full-length transcripts and differential splicing of mRNA [23], particularly for polyploid species or species lacking a high-quality reference transcriptome [24]. As a third-generation sequencing technique, single-molecule real-time (SMRT) sequencing can overcome these limitations by generating long reads without further assembly. It is highly suitable for isoform discovery, in de novo transcriptome analysis [25]. Isoform sequencing (ISO-seq) from PacBio dependent on the SMRT sequencing platform has been used to analyze full-length transcriptomes in sorghum [23], maize [25], rice [26], *Pteris vittata* [27], and pepper [28].

Roots act as the first barrier to Cd exposure for plants grown in Cd-contaminated soils. Identifying Cd-inducible genes in Italian ryegrass roots can deepen our understanding of molecular regulatory pathways in response to Cd stress. In this study, we constructed a de novo full-length transcriptomic database for Italian ryegrass root with the ISO-seq technique. Integrated with RNA-seq, we identified the differentially expressed genes (DEGs) and analyzed the regulated networks related to Cd tolerance and translation. The function of a DEG *LmAUX1* was validated in *A. thaliana*. The overexpression of this gene significantly decreases plant Cd tolerance and enhances plant Cd concentration. Thus, it is possible that this gene could be used for regulating Cd accumulation in Italian ryegrass.

## 2. Results

### 2.1. Assembly of the Full-Length Reference Transcriptomic Database of Italian Ryegrass

**Figure 1:**
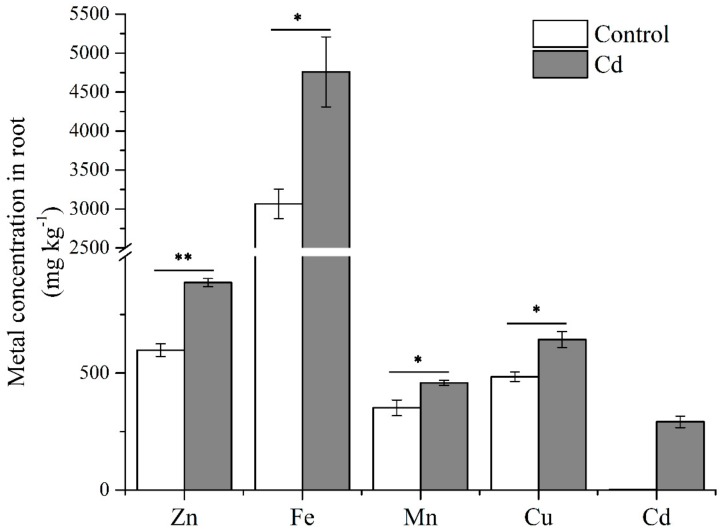
Metal concentrations of Italian ryegrass roots. Data are means ±SE (n = 3). Asterisks indicate significant differences (* *p* < 0.05 and ** *p* < 0.01 by Duncan’s test).

Roots are the first organ to contact Cd stress. To identify gene comportments that foremost regulated plant Cd uptake and tolerance, we treated Italian ryegrass with 50 µmol L^−1^ Cd for six hours. Metal concentrations of Italian ryegrass roots were significant affected by Cd (Figure 1). The concentrations of Zn, Fe, Mn, and Cu were significantly increased, and the Cd concentration reached 291.60 mg k^−1^. To profile the transcriptomic information, total RNA of Italian ryegrass roots was extracted after a six-hour treatment with Cd, and three control and three treated samples were then combined into one sample in equal amounts. The poly(A) RNA was enriched from the mixed RNA sample. Subsequently, according to the results of the pre-experiment, the length of Italian ryegrass mRNA was mostly enriched in a range less than 4 kb (Figure S1). We prepared two overlapping cDNA libraries with insert fragments of 1–4 kb and 3–10 kb, and 20 cycles LD-PCR cycles for a 3–10 kb library were amplified (1–4 kb library, 16 cycles). A library was sequenced by a SMRT cell with the PacBio Sequel platform (Pacific Biosciences, Menlo Park, CA, USA). In summary, we obtained 415,985 and 386,341 reads of inserts for the 1–4 kb library and 3–10 kb library, respectively. Furthermore, 69.06% and 40.47% of them were full-length reads, which had poly(A) tail signals, 5′ adaptor sequences, and 3′ adapter sequences. The average full-length non-chimeric (FLNC) reads were 1711 and 3540 base pairs (bps) for the 1–4 kb library and 3–10 kb library, respectively (Table S1). Usually, the FLNC reads of each cDNA library contain repetitive isoforms. According to the isoform-level clustering algorithm (ICE) analysis, the FLNC reads were aligned, and similarity sequences were assigned to a cluster. Each cluster was identified as a uniform isoform. The isoform sequences were then polished and integrated with non-full-length non-chimeric reads. Isoforms with a predicted accuracy of >99% were considered high-quality isoforms, and others were regarded as low-quality. The average lengths of consensus isoform reads were modified to 1756 and 3653 bps in the 1–4 kb and 3–10 kb libraries, respectively (Table S2).

To further improve the accuracy of the PacBio ISO-seq data, we sequenced six samples of the Italian ryegrass root with the same treatment as PacBio ISO-seq on the Illumina Hiseq X Ten platform (Illumina, San Diego, CA, USA) with 150 bp pair-ends. In total, each of RNA-seq samples generated more than 39,340,473 clean reads (Table S3). The low-quality isoforms generated by ICE proofread were corrected with RNA-seq short reads using LSC software (http://augroup.org/LSC/LSC/), and the high-quality isoforms and corrective low-quality isoforms were then combined as whole full-length isoforms. In standard ISO-seq analysis, a transcript may generate different isoforms, and these isoforms may be assigned to different libraries, so we removed redundant isoforms using CD-HIT-EST software (http://www.bioinformatics.org/cd-hit/). Finally, the non-redundant high-quality transcripts were obtained and considered the reference transcriptome with a size of 340 Mb.

### 2.2. Annotation of the Full Length Reference Transcriptome

All isoforms of the reference transcriptome were aligned to the protein and nucleotide databases including NCBI non-redundant proteins (NR), NCBI non-redundant nucleotides (NT), Swiss-Prot, Gene Ontology (GO), Kyoto Encyclopedia of Genes and Genomes (KEGG), and KOG. As shown in Table 1, in total, 146,545 isoforms were annotated; there were 123,344 isoforms annotated in the GO database and 72,725 isoforms annotated in the KEGG database. The best harbored isoforms in the reference transcriptome were in the NR database, and 145,825 isoforms hit in the NR database.

**Figure 2:**
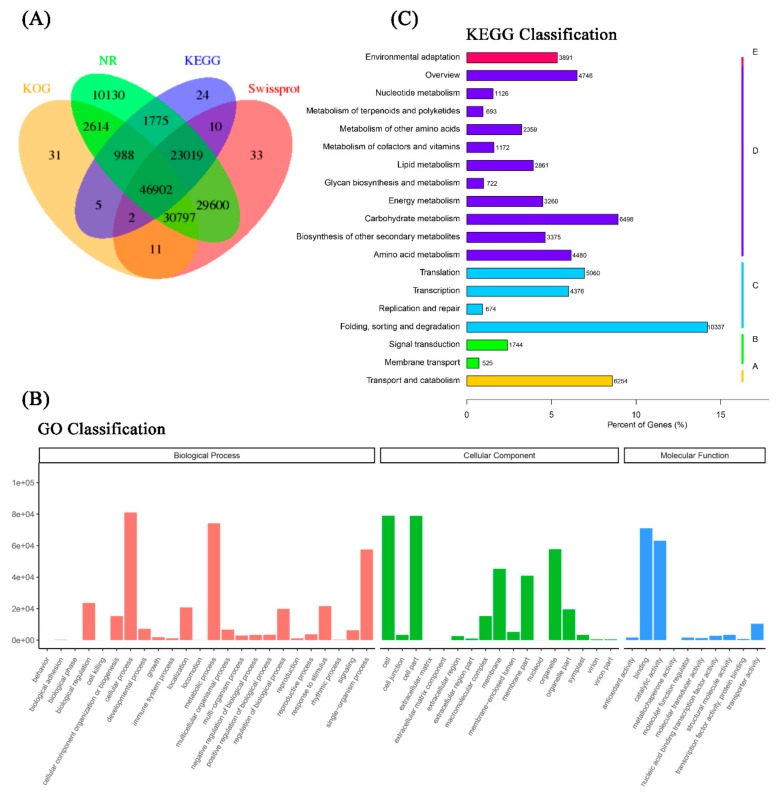
Functional annotation of the full-length reference transcriptome. (**A**) Venn diagram of all isoforms from the reference transcriptome hits of KOG, non-redundant proteins (NR), Kyoto Encyclopedia of Genes and Genomes (KEGG), and SwissProt databases. (**B**) Gene ontology classification of all identified isoforms from the reference transcriptome. (**C**) KEGG classification of all identified isoforms from the reference transcriptome.

Overall, there were 30,797 isoforms mapped in at least three databases, and 46,902 isoforms hit four databases (KOG, NR, KEGG, and Swiss-Prot) (Figure 2A). For GO analysis, the isoforms were annotated in three categories—biological process (BP, 352,144 isoforms), cellular component (CC, 353,049 isoforms), and molecular function (MF, 156,073 isoforms). Within the functional classifications, cellular process (GO:0009987, 81,045 isoforms) and metabolic process (GO:0008152, 74,167 isoforms) were the two most functional terms in BP; cell (GO:0005623, 79,012 isoforms) and cell part (GO:0044464, 78,846 isoforms) were the two most functional terms in CC. Catalytic activity (GO:0003824, 63,137 isoforms) and binding (GO:0005488, 70,972 isoforms) were the most abundant functional terms in MF (Figure 2B). To further profile the pathways that all isoforms take part in, the KEGG Orthology identifiers (KOs) that annotated isoforms enriched in the KEGG database were classified in five metabolic pathways (Hierarchy 1). In cellular process KOs, the isoforms were mainly associated with transport and catabolism (6254 isoforms, 8.6%) pathways. There were 2269 isoforms (3.1%) that took part in environmental information processing KOs. In genetic information processing KOs, there were 20,447 isoforms (28.1%) that participated, and they were mainly focused on folding, sorting, and degradation pathways. More than 43.0% (31,292) isoforms hit for metabolism KOs, which were involved in many pathways, such as carbohydrate metabolism, amino acid metabolism, and global and overview maps. Further, less than 5.4% (3891) of isoforms were categorized into organismal system KOs and were involved in environmental adaptation pathways (Figure 2C).

### 2.3. Identification and Functional Profiles of Differentially Expressed Genes

**Figure 3:**
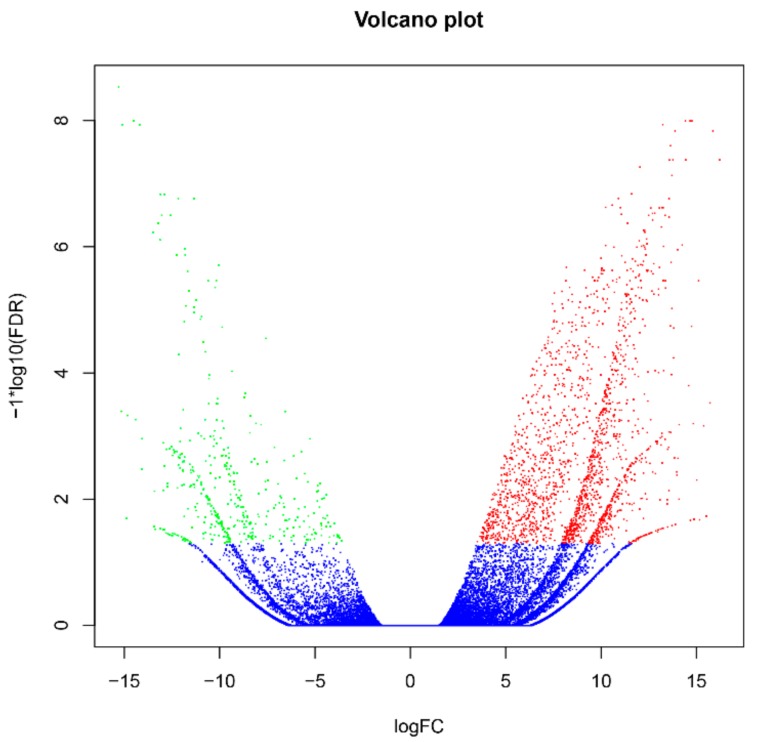
Distribution and expression levels of differentially expressed genes (DEGs) in Italian ryegrass roots exposed or not to cadmium (Cd). The x-axis represents the log_2_ [Fold Change] values under the mean normalized expression of all isoforms (y-axis). Red dots indicate the upregulated DEGs, and green dots represent the down regulated DEGs.

The filtered clean reads from Illumina RNA-seq were mapped to the reference transcriptome generated by PacBio ISO-seq. Of all the reads, 68.73–71.43% were mapped to the reference transcriptome, 9.22–10.29% were uniquely mapped reads, and 98.71–90.78% were multiple aligned reads (Table 2). The expression levels of all isoforms were calculated by RESM software and shown as FPKM values. With the edgeR package, the different expression levels of the isoforms were assessed according to the threshold (FDR values < 0.001). A total of 2367 differentially expressed genes (DEGs) were obtained in response to Cd stress, 1944 DEGs were significantly upregulated, 423 DEGs were significantly downregulated (Figure 3), and relative expression levels of 20 random selected DEGs were further verified using qRT-PCR (Figure S2). Specific primers were designed with Primer Premier 5.0 (PREMIER Biosoft International, Palo Alto, CA, USA) (Table S6).

**Figure 4:**
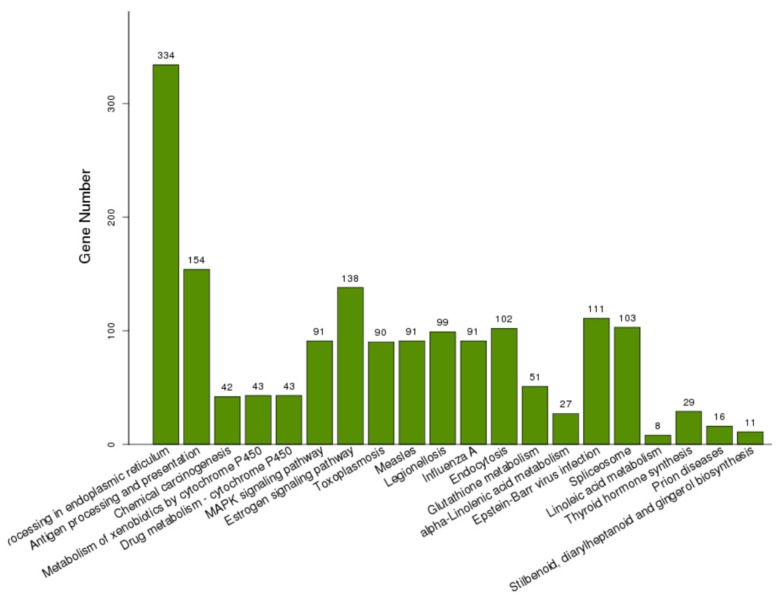
KEGG pathway enrichment analysis based on the DEGs significantly enriched in KOs. The number of above each bar was the number of DEGs enriched in each KO.

To characterize the functions of the DEGs, GO enrichment analysis was performed to categorize potential functions (Supplementary Materials Table S1). The DEGs significantly annotated in the top 20 terms in the BP, MF, and CC categories are shown in Table 3, Table 4 and Table 5. For the BP category, the four most enriched GO terms were associated with oxidation reduction processes (GO:0055114), response to heat (GO:0009408), protein folding (GO:0006457), and response to oxidative stress (GO:0006979). The two terms involved in unfolded protein binding (GO:0051082) and heme binding (GO:0020037) were assigned to the MF category. The extracellular regions (GO:0005576) and vacuoles (GO:0005773) were the two most important terms in the CC category. The DEGs were also subject to KEGG analysis, and 1094 DEGs were assigned to 205 pathways (Supplementary Materials Table S2). The DEGs significantly enriched in the top 20 significant KOs are shown in Figure 4. The highly enriched pathways of DEGs were protein processing in the endoplasmic reticulum (ko04141) and antigen processing and presentation (ko04612). The endocytosis (ko04144) and glutathione metabolism (ko00480) also appeared in the main KEGG pathways of the DEGs.

### 2.4. Functional Validation of LmAUX1 in Response to Cd

To confirm whether these putative genes were associated in plant Cd tolerance, we cloned a significantly downregulated gene, according to the blastp results, and named it *LmAUX1*. We hypothesized that it may affect plant Cd uptake and distribution. The predicted CDS of this gene (Multiflorum_1-4k_c11285_f6p6_2149) was isolated and over-expressed in *A. thaliana* (Col-0 and *aux1-7* mutant).

**Figure 5:**
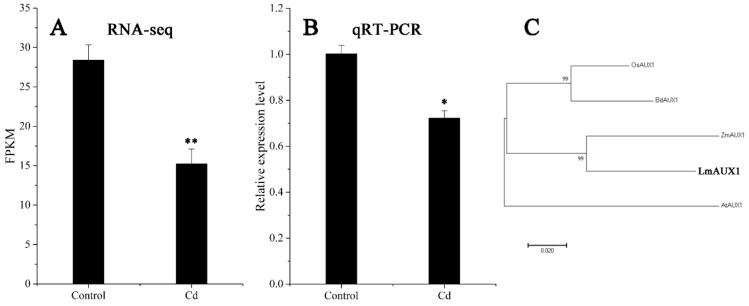
Expression level and phylogenetic tree analysis of *AUX1*. (**A**) Expression level of *LmAUX1* calculated by RNA-seq. Data are means ± SE (n = 3). Asterisks indicate significant differences (** *p* < 0.01 by Duncan’s test). (**B**) Relative expression level of *LmAUX1* verified by qRT-PCR. Data are means ± SE (n = 4). Asterisks indicate significant differences (* *p* < 0.05 by Duncan’s test). (**C**) Phylogenetic tree of AUX1 proteins: the phylogenetic tree was constructed from an alignment of AUX1 proteins from five different plant species by neighbor-joining methods with bootstrapping analysis (1000 replicates), AtAUX1 (*Arabidopsis thaliana*, AT2G38120), BdAXU1 (*Brachypodium distachyon*, Bradi2g55340), LmAUX1 (*Lolium multiflorum*, Multiflorum_1-4k_c11285_f6p6_2149), OsAUX1 (*Oryza sativa*, Os01g63770), and ZmAXU1 (*Zea mays*, PWZ17958).

**Figure 6:**
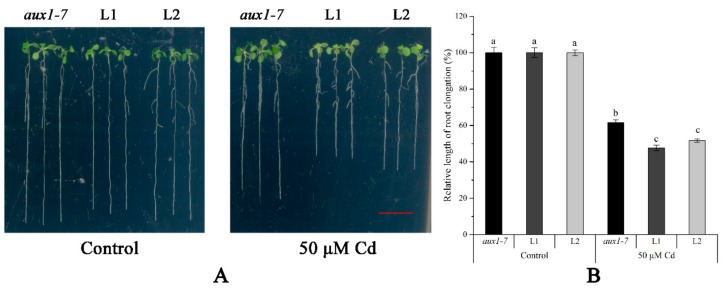
The Cd tolerance experiment on plates of *LmAUX1*. *LmAUX1* was transformed into the *Arabidopsis thaliana aux1-7* mutant with the *CaMV35S* promoter, and two lines (L1 and L2) were used for the experiment. (**A**) Phenotype of plants grown in 1/2 MS medium with and without 50 μM Cd for 4–5 d. Scale bar = 1 cm. (**B**) Relative length of root elongation. Data are means ± SE (n ≥ 15); different letters above the bars were significantly different at *p* < 0.05 (Duncan’s test).

**Figure 7:**
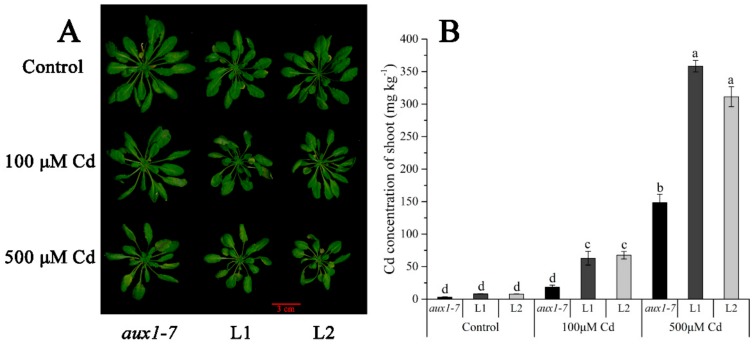
Cd translocated experiment in soil of *LmAUX1*. *LmAUX1* was transformed into the *Arabidopsis thaliana aux1-7* mutant with the *CaMV35S* promoter, and two lines (L1 and L2) were used for the experiment. (**A**) Phenotype of plants grown in soils irrigated with 0, 100, or 500 μM Cd solution for 3–4 weeks. (**B**) The Cd concentration of shoot. Data are means ± SE (n = 3); different letters above the bars are significantly different at *p* < 0.05 (Duncan’s test).

The FPKM values obtained from RNA-seq and relative expression levels verified by qRT-PCR of the DEG (Multiflorum_1-4k_c11285_f6p6_2149) are shown in Figure 5A,B, respectively. According to the analysis of the phylogenetic tree, it was a homologue gene of *AUX1* in *A. thaliana*, *Brachypodium distachyon*, *Zea mays*, and rice (Figure 5C). Multiple transgenic lines of *A. thaliana* (Col-0 ecotype and *aux1-7* mutant) were generated by the T-DNA-inserted fragment harboring *35S:lmAUX1*. Two independent transgenic lines appeared as Cd-tolerance trails, respectively. The T3 generations of transgenic lines, the *aux1-7* mutant, and Col-0 were treated with and without 50 µM CdCl_2_ in 1/2 MS medium (Figure 6A and Figure S3A), and the results clearly showed that the relative length of root elongation in the transgenic lines was significantly lower than those of the *aux1-7* mutant and Col-0 (Figure 6B and Figure S3B). Col-0, the *aux1-7* mutant, and the overexpression lines were grown in soils irrigated with water or with a 100 or 500 µM CdCl_2_ solution, respectively (Figure 7A), and the shoot Cd concentration of transgenic lines was significant increased compared to the *aux1-7* mutant (Figure 7B) and Col-0 (Figure S4). The shoot Zn, Mn, and Cu concentrations of transgenic lines was not significant affected compared to the *aux1-7* mutant and Col-0, but the Fe concentration of the transgenic lines was significant changed compared to the *aux1-7* mutant (Figure S5) and Col-0 (Figure S6), except *aux1-7* and its overexpression lines (L1 and L2) treated with 500 µM Cd.

### 2.5. Alternative Splicing Identification

**Figure 8:**
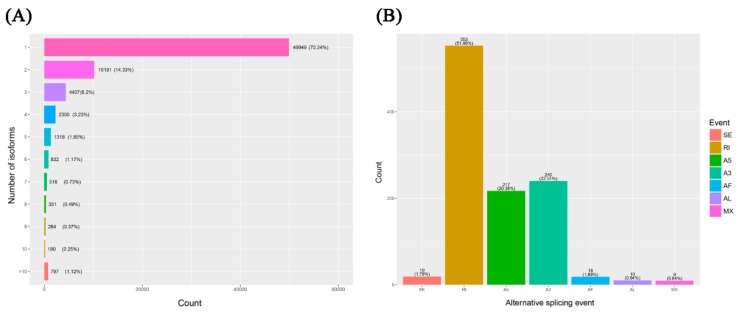
Alternative splicing (AS) analysis of the *Lolium multiflorum* full-length transcriptome using ISO-seq. (**A**) Distribution of isoform numbers for UniTransModels. (**B**) Numbers of different AS events detected in full-length transcriptome. SE: skipping exon; RI: retained intron; A5: alternative 5′ splice-site; A3: alternative 3′ splice-site; AF: alternative first exon; AL: alternative last exon; MX: mutually exclusive exons.

The inherent advantage of PacBio ISO-seq makes it possible to understand the complexity of alternative splicing (AS) at the whole transcriptome scale even without a reference genome. In this study, using the full-length reference transcriptomic database, we reconstructed the unique models of all transcripts by cogent software, named full-length UniTransModels. Transcript isoforms of Italian ryegrass were clustered by mapping individual ISO-seq consensus isoforms back to the reconstructed full-length UniTransModels. In summary, a total of 29.76% of the UniTransModels had more than one isoform, and almost half of the UniTransModels (14.33%) had two isoforms; there were still some UniTransModels with more than 10 isoforms (797, 1.12%) (Figure 8A). The different types of AS events were assessed based on the UniTransModels as the references instead of canonical genome-based AS events. Retained introns (RIs) were the majority of AS events, and together with alternative 5′ end or 3′ end AS events, these three types of AS events constituted more than 90% of detected events (Figure 8B).

By aligning Illumina short reads to transcript models (UniTransModels), we could further validate the reliability of isoforms detected by the pipeline without a reference genome, and different AS events of some isoforms in UniTransModels in control and Cd stress treatments are illustrated in Figure S7.

## 3. Discussion

In the past decade, next-generation sequencing (NGS) technology has been widely used for genetic discovery and research [29], and the well-known short-read RNA-seq has become an integral part in evaluating whole RNA expression patterns [30]. However, the sequenced fragments of NGS are usually between 50 and 300 bp, and this was insufficient in the accurate reconstruction and reliable expression estimation of transcript variants, especially for species with low-quality genomes or even without reference genomes [31]. The studies of the species that lack reliable genetic information were seriously restricted in the precise understanding of the genetic diversity and whole expression patterns. Single-molecule long-read sequencing, which was a third sequencing technique, can identify the full structure of individual transcripts by full-length sequencing, which can uniquely reveal the complexity of the transcriptome [23,25].

Italian ryegrass is one of the most widely used forage species in the world [32]. For now, forage grasses are not only used for feedstuff and soil stability enhancer, but also potentially used for biofuel and bioremediation activities [5]. Herbaceous crops may play an important role in sustainable bioenergy feedstock in the future [33]. Italian ryegrass can provide substantial biomass feedstocks due to its high yields and rapid growth. A report indicated that Italian ryegrass was a high-potential bioethanol resource with a higher yield of bioethanol (84.6%) compared to switchgrass (77.7%) and silvergrass (64.3%) [6]. Molecular breeding in crops has shown promise in increasing yield and multiple stress in a changing climate and environment, and it may improve the desirable characteristics in the breeding of Italian ryegrass with molecular biology methods. Transcriptomic analyses are important for understanding gene function. The current knowledge about ryegrass is mainly dependent on gene expression sequenced NGS technologies [34,35]. As a result, the fragmentation of RNA information cannot fully characterize the complexity and diversity of the transcriptome.

In this study, we constructed the transcriptome of Italian ryegrass root using PacBio ISO-seq. We aimed to obtain full and accurate transcriptome information about ryegrass so as to unveil the various responsive networks under Cd stress. Given our pre-experiment, we constructed two overlapped libraries (inserted sizes of 1–4 kb and 3–10 kb), rather than the normal libraries, i.e., 1–4 kb and 4–10 kb libraries. In summary, we obtained 8.12 and 5.52 GB of raw data, respectively (Table S4). According to the SMRT standard protocol procedures, the raw data were then further filtered (Table S5 and Figure S8). The results of quality control indicated that, instead of using the multiple size-fractionated libraries method [25], shortages in the sequencing data between 3 and 4 kb were supplemented, and the overlapped strategy avoided the biases that shorter fragments of a unique library sequence were regnant (Figure S9). With further classification, clustering, polishing, and comprehensive filter procedures as well as integration with Illumina RNA-seq data, we obtained a 340 Mb full length reference transcriptome, and 146,545 isoforms were annotated with different protein and nucleotide databases. The various functions of Italian ryegrass transcripts were thus unveiled.

RNA-seq is one of the most well-known transcriptomic research pipelines. It is useful for understanding the expressed patterns and for identifying genes, be they of model or non-model species [36]. Cadmium is a toxic heavy metal element, causing serious harm to almost all plants, animals, and humans [8]. Understanding the regulatory mechanisms of genes controlled under Cd may contribute to precise breeding with safer forage or new uses of Italian ryegrass in phytoremediation. In this work, nine-day Italian ryegrass seedlings were treated with and without Cd treatment. After six hours, the concentrations of heavy metals were significant affected by Cd. The mRNAs of six Italian ryegrass roots with and without Cd treatment were sequenced with RNA-seq. Over 60 GB of raw data were obtained. Heat map analysis indicated a correlation of all samples, which were clustered as the control group and the Cd group (Figure S10). The data were aligned with the reference transcriptome generated by PacBio ISO-seq, and a large number of DEGs were generated, which provided valuable information on the transcriptional regulation of genes in responses to Cd stress in Italian ryegrass.

Gene Ontology (GO) enrichment analysis enhanced our understanding of DEGs. The DEGs of biological processes involved oxidation–reduction processes, protein folding, and responses to oxidative stress, which was present in the highest proportion. Cadmium can lead to a rise in ROSs and disturb redox homeostasis [37]. Overproduction of ROS can generate oxidative stress, causing protein carbonylation, which can lead to misfolded or inactivated proteins [38]. In addition, some DEGs are involved in responses to heat terms. Heat Shock Proteins (HSPs) play a crucial role in plant tolerance to multiple stress [39]. Some HSP/chaperone genes have been reported to contribute to the positive enhancement of plant Cd tolerance [20,40,41]. HSPs can also act as molecular chaperones to process the folding, assembly, translocation, and degradation of matched proteins [42]. For example, the HSP chaperones are responsible for the folding of some nascent proteins to alleviate endoplasmic reticulum (ER) stress [43], including kinases [44], transcription factors [45], and transporters [46]. Previous transcriptomic research on Cd-treated plants has also emphasized that these biological processes all serve as important functions in adaptation to biotic and abiotic stresses, but these are not limited to Cd stress [21,47]. In addition, some DEGs have been classified as contributing to cellular components, such as extracellular regions, vacuoles, and cell walls in common with other RNA-seq analyses [48]. Additionally, few Cd-responsive genes in Italian ryegrass have been cloned and characterized. Therefore, a list of DEGs as candidate genes could be helpful for further studies (Supplementary Materials Table S3).

In previous studies, the homeostasis of auxin was found to be disturbed by Cd in *A. thaliana* [49,50]. Additionally, our previous study also showed that Cd interfered with the auxin homeostasis (Figures S11 and S12), and the loss of function of the auxin transporter enhanced plant Cd tolerance (Figure S13). We transformed the *lmAUX1* into the *aux1-7* mutant, and the tropic movement of roots was regained (Figure S14). In the Cd tolerance on plates, the relative length of root elongation was significant inhibited. Additionally, soil experiments showed that the Cd concentration of shoots was significantly increased. These results indicate that *LmAUX1* can regulate plant Cd tolerance and accumulation. In Italian ryegrass, the relative expression level of *lmAUX1* was downregulated under Cd stress, so it is suggested that Italian ryegrass enhanced self-tolerance by decreasing the expression level of *LmAUX1*.

Alternative splicing (AS) is an important post-transcriptional regulatory mechanism for the diversity of transcriptomes and proteomes [51]. Recently, the versatile roles of AS in plants have been further clarified, such as the salt stress tolerance in *A. thaliana* and the mineral nutrient homeostasis in rice. In *A. thaliana,* the U1 spliceosomal protein, AtU1A, is responsible for recognizing the 5′ splice site in the initial step of pre-mRNA splicing. The 5′ splice site in the *Arabidopsis aconitase* (*ACO1*) pre-mRNA can be sequentially recognized by the U1 snRNA that is associated with the AtU1A protein. Splicing of *ACO1* pre-mRNA is important for maintaining proper ROS levels in the cell and for contributing to salt tolerance [52]. Ser/Arg (SR)-rich proteins interact with pre-mRNA sequences and splicing factors during spliceosome assembly in order to perform essential functions in constitutive and AS. In rice, three SR protein-encoding genes (*SR40*, *SCL57*, and *SCL25*) regulate P uptake and remobilization between leaves and shoots of rice [53]. However, these studies have mainly focused on model plants using NGS techniques. For polyploid species or un-sequenced species, it is difficult to accurately identify transcript isoforms [24]. PacBio ISO-seq is a more reliable and superior strategy to understand the whole transcriptomes. It can directly generate accurate AS isoforms, rather than short read sequencing, to annotate a novel whole-genome assembly [54]. Here, we characterized AS in autotetraploid Italian ryegrass using PacBio ISO-seq. In common with results with respect to cotton [24], rice [26], strawberry [29], and switchgrass [55], the retained intron events contributed the most AS events. In addition, the percentage of unique isoforms is greater than those of other species; it appeared that polyploidy also played an important role in increasing the transcript diversity of Italian ryegrass [56]. Our results only scratch the surface of the AS in Italian ryegrass under Cd stress without the reference genome, and we expect to reveal more AS transcript-harboring genes under multiple stresses in the future.

## 4. Materials and Methods

### 4.1. Plant Materials, Growth Conditions, and Treatment

A Cd-tolerant ryegrass cultivar (“IdyII”) was chosen for this study [57]. The Italian ryegrass seeds were treated with 50% H_2_SO_4_ for 5 min, washed with deionized water thoroughly, subsequently disinfected with 30% NaClO for 15 min, and then rinsed with deionized water again. After that, the seeds were preliminarily germinated on a net floating on deionized water at 25 °C in the dark. Three days later, the germinated seeds were cultivated in a greenhouse with a day/night temperature of 25/20 °C, 65 ± 5% relative humidity, and a 12 h light/dark cycle with 150 µmol m^−2^ s^−1^ light intensity for two days. The seedlings with a uniform height of about 4 cm were transferred to a 1 L black plastic beaker filled with a 1/4 strength modified Hoagland solution with the following composition: 1.5 mmol L^−1^ KNO_3_, 1.0 mmol L^−1^ Ca(NO_3_)·4H_2_O, 0.5 mmol L^−1^ NH_4_H_2_PO_4_, 0.25 mmol L^−1^ MgSO_4_·H_2_O, 50 µmol L^−1^ KCl, 25 µmol L^−1^ H_3_BO_3_, 2.0 µmol L^−1^ MnSO_4_·H_2_O, 2.0 µmol L^−1^ ZnSO_4_·7H_2_O, 0.5 µmol L^−1^ CuSO_4_·5H_2_O, 0.5 µmol L^−1^ (NH4)_6_MO_7_O_24_·4H_2_O, and 20 µmol L^−1^ Fe(II)-EDTA-Na_2_. The pH value was adjusted to 6.5, and the nutrient solution was renewed every 3 d. Seedlings were continuously cultivated in the greenhouse. After pre-culture for 9 d, plants were treated with and without 50 µmol L^−1^ Cd supplied as CdCl_2_ for 6 h. There were three biological replicates for the assays. A black beaker containing seven seedlings represented a biological replicate. From each beaker, two seedlings used for PacBio ISO-seq and Illumina RNA-seq were chosen, respectively. Other seedlings were used for the determination of metal concentrations.

### 4.2. RNA Isolation, RNA-seq, and PacBio Full-Length ISO-SeqLibrary Preparation and Sequencing

Total RNAs of root samples were extracted using TRIzol (Invitrogen™, Life Technologies, Carlsbad CA, USA), and residual DNA was removed with RNase-free DNase I (New England BioLabs, Ipswich, MA, USA) according to the manufacturer’s instructions. The total RNA was quantified and assessed with an Agilent Bioanalyzer 2100 (Agilent Technologies, Palo Alto, CA, USA), and the RNA integrity numbers (RIN) of all samples were greater than 9.0. Total RNA samples were sent to Berry Genomics (http://www.berrygenomics.com) for sequencing.

The RNA-seq libraries were constructed using the TruSeq RNA Sample Prep Kit (Illumina, San Diego, CA, USA) as per the manufacturer’s instructions. The libraries were sequenced using Illumina Hiseq X Ten (Illumina, San Diego, CA, USA) as 150 bp paired-end reads.

For the PacBio full-length ISO-seq libraries, two equal mixed RNAs (2 μg, mixed with equal three controls and three-treatment total RNAs) were used for 1–4 and 3–10 kb cDNA library construction, respectively. Poly(A) RNA was isolated using the Poly(A) Purist^TM^ Kit (Ambion, Inc., Austin, TX, USA)). RNA was reverse-transcribed into cDNA using the SMARTer™ PCR cDNA Synthesis Kit (Clontech Laboratories Inc., Palo Alto, California, USA). In PCR amplification, for 1–4 kb library construction, the LD-PCR included 16 cycles, and for 3–10 kb library, the LD-PCR included 20 cycles. The 1–4 and 3–10 kb cDNA fragments were generated by BluePippin size selection (Sage Science, Beverly, MA, USA). The size selected cDNA products were applied to constructed SMRTbell Template libraries (SMRTBell Template Prep Kit, Pacific Biosciences, Menlo Park, CA, USA). Each library used one SMRT cell sequenced on a PacBio sequel platform (Pacific Biosciences, Menlo Park, CA, USA). 

### 4.3. Assembly of Reference Transcriptomic Database

Raw short reads of Illumina RNA-seq were filtered by removing reads containing adaptors, reads containing poly-N (>10%), and low-quality reads. Q20, Q30, GC-contents, and sequence duplication levels of the clean data were calculated. Clean reads were used for next analysis.

The standard protocol of ISO-seq (SMRT Analysis 2.3) was used to process the raw PacBio full-length ISO-seq data. Raw long reads were filtered, and the reads for which length was no more than 50 bp and of which accuracy was less than 0.75 were removed. The reads of inserts (ROIs) were obtained from the circular consensus sequences (CCS). After examining for 5′ and 3′ adaptors and poly(A) signals, full-length and non-full-length cDNA reads were defined, and chimeric reads were removed, which included sequencing primers. According to the isoform-level clustering algorithm (ICE), the full-length non-chimeric reads were aligned, and similarity sequences were assigned to a cluster. Each cluster was identified as a uniform isoform. Non-full-length cDNA reads were then applied to polish each cluster. The isoform sequences (with a predicted accuracy of >99%) were considered high-quality isoforms, and others were regarded as low-quality isoforms. The Illumina short reads were used to improve the low-quality isoform accuracies by LSC (LSC 2.0, http://augroup.org/LSC/LSC)). The modified low-quality isoforms and high-quality isoforms were combined as high-quality full-length transcripts. Finally, the redundancies were moved using CD-HIT-EST (CD-HIT-EST 4.6, http://www.bioinformatics.org/cd-hit, parameterization: c = 0.99, T = 6, G = 1, U = 10, s = 0.999, d = 40, and p = 1) to obtain non-redundant high-quality transcripts. The transcripts were considered the reference transcriptome and used for further analysis.

### 4.4. Annotation of Gene Function

To understand the functions of the reference transcriptome, the protein-coding sequences (CDS) of all transcripts were predicted by TransDecoder (Transdecoder 3.0.0, https://github.com/TransDecoder) at the default setting. The coding likelihood scores of six open reading frames (ORFs) with a length of coding protein sequences of more than 100 amino acids (aa) in a transcript were counted, and the highest score of ORFs was retained as the CDS. If a candidate ORF was found fully encapsulated by the coordinates of another candidate ORF, the longer one was reported [58]. All predicted CDSs were aligned to protein and nucleotide databases (NCBI non-redundant protein (NR), NCBI non-redundant nucleotide (NT), Swiss-Prot, Gene Ontology (GO), KEGG, and KOG) using blast.

### 4.5. Quantification of Gene Expression Levels

All clean reads of Illumina RNA-seq corresponding to each pooled sample were mapped against the assembled reference transcriptome using bowtie 2 (V2.1.0). The FPMK (fragments per Kilobase Million) values of each isoform were computed for each pooled sample and normalized using the RPKM (RSEM 1.2.15) [59].

### 4.6. Identification and Function Assessment of DEGs

EdgeR package (edgeR 3.14.0) was used to identify differentially expressed genes (DEGs) between the control and Cd treatment. Significant DEGs were identified with the threshold of FDR < 0.001 and log_2_ (FoldChange) > 1.

The GO enrichment analysis was implemented to assess the functions of DEGs using the topGO R package (topGO 2.24.0). The KEGG pathways were applied to enrich the DEGs to different biochemical metabolic pathways and signal transduction pathways with KOBAS software (KOBAS 2.0).

### 4.7. Validation of DEGs with qRT-PCR

The relative expression levels of 20 randomly selected DEGs were validated with qRT-PCR. New total RNAs were isolated from the plants re-cultivated as were previous samples used for sequencing. The cDNA was synthesized with the HiScript^®^ Q RT SuperMix for qPCR (Vazyme, #R223-1, Nanjing, China). The qRT-PCR was carried out using the ChamQTM SYBR^®^ Master Mix (Vazyme, #Q311-02/03, Nanjing, China) in the QuantStudio 5 Real-Time PCR System (Applied Biosystems, Foster City, CA, USA). All samples were normalized with the reference gene *LmeEF1A(s)*, calculated using the 2^−ΔΔCt^ method, and shown as mean ±SE. Four biological replicates were performed.

### 4.8. Vector Construction and Ectopic Overexpression of LmAUX1 in Arabidopsis Thaliana

To reveal the functions controlled by the genes of Italian ryegrass response to Cd stress, a DEG (Multiflorum_1-4k_c11285_f6p6_2149) was selected for functional characterization. A blastp search in the National Center for Biotechnology Information (NCBI) with predicted protein coding sequences of this gene as the default setting indicated that it coded an auxin transporter. Thus, it was named *LmAUX1*. The RNA-seq result showed that the relative expression level of this gene was significantly decreased in response to Cd stress, and this was validated with qRT-PCR. The phylogenetic tree, based on amino acid sequences of predicted protein coding sequences and AUX1 amino acid sequences of several other plant species, was constructed using MEGA 7 software (Molecular Evolutionary Genetics Analysis 7.0 software). Bootstrapping analysis was processed as the neighbor-joining method (1000 replicates).

The predicted CDSs of *LmAUX1* were cloned into the pCAMBIA1305-eGFP vector with the *CaMV35s* promoter. The constructed vector was chemically transformed into the *Agrobacterium tumefaciens* strain “EHA105,” and the target gene was then transformed into *A. thaliana* (*LmAUX1* was transformed into both the Col-0 ecotype and the *aux1-7* mutant, respectively) by the floral dip method. Positive transgenic lines were screened with 25 mg L^−1^ hygromycin on the half-strength Murashige and Skoog solid medium supplied with 1% sucrose and 0.8% agar (pH 5.8) (1/2 MS medium). More than 15 transgenic lines were obtained, and two T3 lines of them were used for functional characterization. The *LmAUX1* overexpression lines transformed into *aux1-7* mutants named L1 and L2, and *LmAUX1* overexpression lines were transformed into Col-0 ecotypes named OE1 and OE2.

For Cd tolerance experiments on plates, seeds were surface-sterilized with 10% NaClO for 15 min and plated on 1/2 MS medium. The seeds were vernalized for two days in darkness at 4 °C and then cultivated in a growth chamber with a 16/8 h light/dark cycle and a temperature of 23/20 °C for the day/night cycle. After 4–5 days, seedlings were transferred to 1/2 MS medium with and without 50 µM CdCl_2_. After five days, the seedlings were photographed, and the length of primary root elongation was measured using the ImageJ software. The experiments were repeated twice.

For Cd transferred experiments in soil, Col-0, *aux1-7*, and *LmAUX1* overexpression lines (L1, L2, OE1, and OE2) were grown in soil under a long-day photoperiod (16/8 h light/dark) for two weeks. Subsequently, the plants were grown on soils irrigated with a 0, 100, or 500 μM Cd solution every two or three day for 3–4 weeks. 

### 4.9. Determination of Metal Concentrations

The samples were dried at 70 °C for 3 d and then subjected to with mixed acid (HNO3 + HClO4 (85:15, *v/v*)) using a DigiBlock ED54-iTouch Digester (LabTech, Beijing, China). The samples were completely digested at 170 °C until they turned white; subsequently, the residual acid was volatilized, and all samples were re-dissolved in 10 mL 2.5% HNO3. The concentrations of metals were determined using am inductively coupled plasma optical emission spectrometer (ICP-OES, Perkin Elmer Optima 8000).

### 4.10. Microscopic Imaging of GUS Staining and Fluorescence of DII-VENUS

GUS staining was carried out according to the method reported in Hu et al. [50]. GUS activity in primary root apices in 5-day-old *DR5::GUS* seedlings treated with 0, 25, 50, 100 μM Cd for 3–4 days on 1/2 MS plates. GUS-stained images were observed under a stereo microscope (ZEISS Stemi 2000-C) and photographed by a CCD camera (Canon PowerShot A620).

Confocal microscopy was performed using a confocal laser scanning microscope (PerkinElmer, Waltham, MA, USA, UltraVIEW^®^ VoX) according to the manufacturer’s instructions, excitation and emission wavelengths were 488 to 520 nm for DII-VENUS. Auxin signaling level in primary root apices in five-day-old *Arabidopsis thaliana DII-VENUS* seedlings treated with 0, 25, 50, 100 μM Cd for 3–4 days on 1/2 MS plates.

### 4.11. Alternative Splicing Identification

The reference transcriptome was re-constructed using Cogent (coding genome reconstruction tool, Cogent v1.4), and a set of transcripts models was then generated and named UniTransModels [60]. All isoforms of the reference transcriptome (transcripts before Cogent reconstruction) were then aligned to UniTransModels with GMAP software (GMAP v2017-06-20). At the same time, a gff file was generated, including the coordinate information of isoforms with respect to the UniTransModels. The transcripts were mapped to the UniTransModels and used for the splicing junctions assessed. The same splicing junctions of the transcripts were collapsed together, and these transcripts with different splicing junctions were identified as transcriptional isoforms of UniTransModels. Additionally, alternative splicing (AS) events were detected with the SUPPA pipeline (https://bitbucket.org/regulatorygenomicsupf/suppa/src/master/) using default settings [61].

The AS events were shown by sashimi plots generated using the IGV browser (IGV 2.5) with bam files and a gff file [62]. The bam files were obtained by mapping the Illumina short reads against UniTransModels using TopHat (TopHat 2.0.4). The FASTA file of the UniTransModels was then imported into IGV as a reference, and the corresponding mapped bam files and isoform gff files were loaded into IGV as tracks. After that, the sashimi plots were obtained from the bam file track. In the opened sashimi plot, the red peaks and junctions are from the Illumina bam file and the blue isoform bars are from the gff file.

### 4.12. Accession Numbers

The raw sequence data sequenced in this study have been deposited in the Genome Sequence Archive in the BIG Data Centre, Beijing Institute of Genomics (BIG), the Chinese Academy of Sciences (GSA, http://bigd.big.ac.cn/gsa), under accession number CRA001799.

## 5. Conclusions

In summary, according to the analyses of PacBio long read ISO-seq and Illumina short read RNA-seq data, we constructed a high-quality Italian ryegrass reference transcriptome and unveiled a comprehensive picture of the regulatory networks in Italian ryegrass under Cd stress. We then characterized a DEG (*LmAUX1*) expressed in *A. thaliana*, which significantly enhanced plant Cd accumulation. It is possible that this gene can be used to breed a high-Cd-accumulation Italian ryegrass cultivar. This work deepens our understanding of the mechanism of Italian ryegrass in response to Cd stress and contributes to its gene identification and molecular breeding.

## Figures and Tables

**Table 1:** Information of function annotation.

Database	Annotated Number	Length 0–<1000	Length 1000–<2000	Length 2000–<3000	Length 3000–<6000	Length ≥6000
GO	123,344	5759	59,645	21,204	33,834	2902
KEGG	72,725	3579	38,770	11,890	16,983	1503
KOG	81,350	3319	41,539	13,234	21,480	1778
NR	145,825	7126	72,604	24,372	38,526	3197
NT	139,068	6697	68,837	23,560	36,922	3052
Swiss-Prot	130,374	5837	64,719	22,006	34,869	2943
All	146,545	7269	72,943	24,448	38,678	3207

**Table 2:** Information of RNA-seq clean reads mapped with the reference transcriptome.

Sample Name	Total Reads	Mapped Reads (%)	Unique Mapped Reads (%)	Multi Mapped Reads (%)
Control-1	96,605,492	66,392,568 (68.73%)	6,831,848 (10.29%)	59,560,720 (89.71%)
Control-2	97,407,032	67,836,176 (69.64%)	6,790,096 (10.01%)	61,046,080 (89.99%)
Control-3	78,680,946	56,203,632 (71.43%)	5,183,088 (9.22%)	51,020,544 (90.78%)
Cd-1	102,165,716	71,120,258 (69.61%)	7,203,654 (10.13%)	63,916,604 (89.87%)
Cd-2	88,241,902	61,102,730 (69.24%)	6,008,134 (9.83%)	55,094,596 (90.17%)
Cd-3	89,834,568	63,034,550 (70.17%)	6,166,310 (9.78%)	56,868,240 (90.22%)

Total Reads: number of clean reads; Unique Mapped Reads (ratio): number of one read mapped to unique site (%); Multi Mapped Reads (ratio): number of one read mapped to multiple sites (%).

**Table 3:** Gene Ontology (GO) analysis of DEGs significantly annotated in the biological process (BP).

NO.	GO.ID	Term	Significant
1	GO:0055114	oxidation-reduction process	221
2	GO:0006457	Protein folding	103
3	GO:0009813	Flavonoid biosynthetic process	38
4	GO:0042744	Hydrogen peroxide catabolic process	19
5	GO:0052696	Flavonoid glucuronidation	32
6	GO:0009992	Cellular water homeostasis	17
7	GO:0015793	Glycerol transport	17
8	GO:0006833	Water transport	18
9	GO:0006979	Response to oxidative stress	82
10	GO:0010041	Response to iron(III) ion	14
11	GO:0006098	pentose-phosphate shunt	8
12	GO:0009414	Response to water deprivation	14
13	GO:0009651	Response to salt stress	34
14	GO:0006024	Glycosaminoglycan biosynthetic process	5
15	GO:0006065	UDP-glucuronate biosynthetic process	5
16	GO:0009408	Response to heat	154
17	GO:0044550	Secondary metabolite biosynthetic process	41
18	GO:0019521	D-gluconate metabolic process	7
19	GO:0006559	L-phenylalanine catabolic process	17
20	GO:0006857	Oligopeptide transport	1

**Table 4:** GO analysis of DEGs significantly annotated in the molecular function (MF).

NO.	GO.ID	Term	Significant
1	GO:0051082	Unfolded protein binding	65
2	GO:0020037	Heme binding	52
3	GO:0003924	GTPase activity	36
4	GO:0015250	Water channel activity	17
5	GO:0015254	Glycerol channel activity	17
6	GO:0004601	Peroxidase activity	23
7	GO:0005525	GTP binding	39
8	GO:0005200	Structural constituent of cytoskeleton	20
9	GO:0004197	cysteine-type endopeptidase activity	15
10	GO:0080043	quercetin_3-O-glucosyltransferase activity	27
11	GO:0080044	quercetin_7-O-glucosyltransferase activity	27
12	GO:0033760	2’-deoxymugineic-acid_2’-dioxygenase activity	14
13	GO:0003979	UDP-glucose_6-dehydrogenase activity	5
14	GO:0004623	phospholipase_A2 activity	7
15	GO:0019904	protein domain specific binding	6
16	GO:0004497	monooxygenase activity	36
17	GO:0016709	oxidoreductase activity, acting on paired donors, with incorporation or reduction	17
18	GO:0045548	Phenylalanine ammonia-lyase activity	17
19	GO:0004616	phosphogluconate dehydrogenase (decarboxylating) activity	8
20	GO:0003700	Transcription factor activity, sequence-specific DNA binding	19

**Table 5:** GO analysis of DEGs significantly annotated in the cellular component (CC).

NO.	GO.ID	Term	Significant
1	GO:0005576	Extracellular region	79
2	GO:0005615	Extracellular space	15
3	GO:0005764	lysosome	15
4	GO:0005773	vacuole	67
5	GO:0005788	Endoplasmic reticulum lumen	21
6	GO:0009505	plant-type cell wall	24
7	GO:0009941	Chloroplast envelope	12
8	GO:0005874	microtubule	17
9	GO:0048046	apoplast	24
10	GO:0009570	Chloroplast stroma	1
11	GO:0046658	Anchored component of plasma membrane	4
12	GO:0005774	Vacuolar membrane	25
13	GO:0009535	Chloroplast thylakoid membrane	5
14	GO:0005777	peroxisome	3
15	GO:0009506	plasmodesma	37
16	GO:0005730	nucleolus	8
17	GO:0048226	Casparian strip	5
18	GO:0008287	Protein serine/threonine phosphatase complex	1
19	GO:0005618	Cell wall	41
20	GO:0031201	SNARE complex	2

## Supplementary Materials

Supplementary materials can be found at https://www.mdpi.com/1422-0067/21/3/1067/s1.
