# Supplementary figures and images for: Full-Length Transcriptome Assembly of Italian Ryegrass Root Integrated with RNA-Seq to Identify Genes in Response to Plant Cadmium Stress
Source: Int J Mol Sci. 2020 Feb 6;21(3):1067. doi: 10.3390/ijms21031067 (PMC7037684; doi:10.3390/ijms21031067)

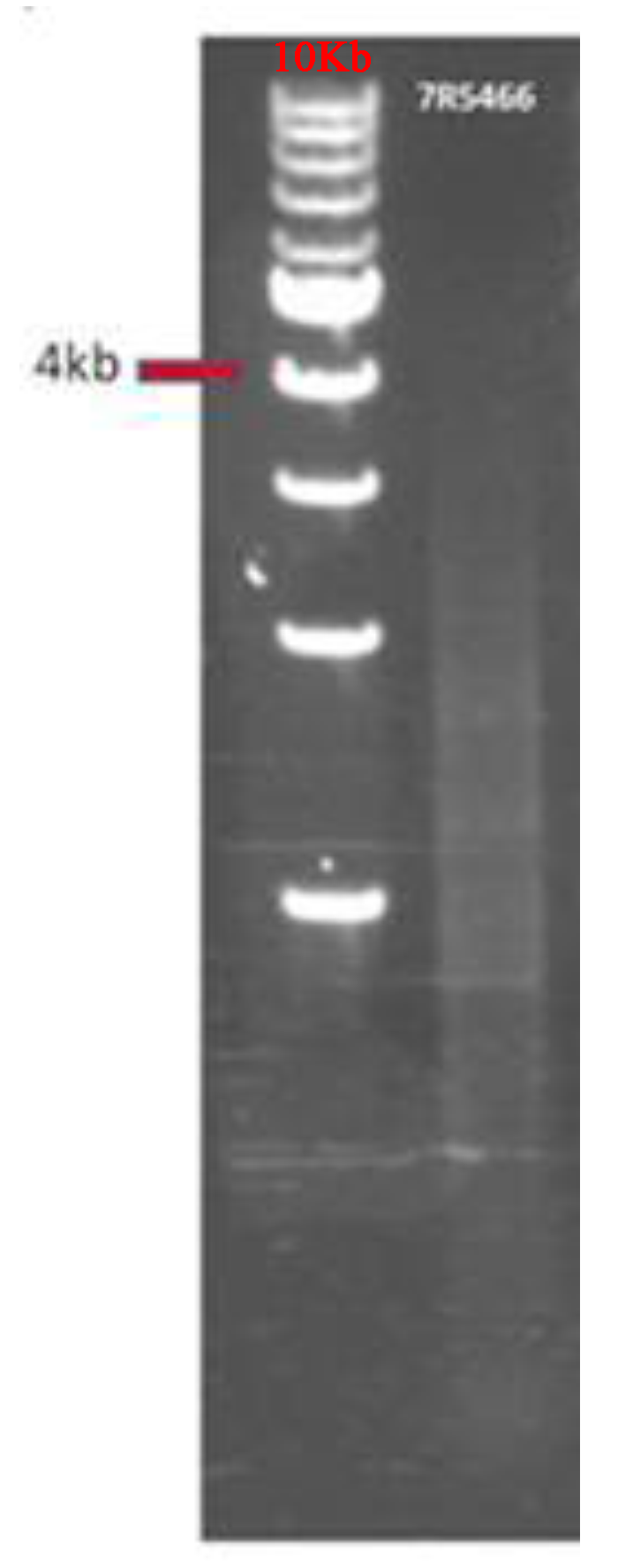

Supplement: Supplementary file 1 [file ijms-21-01067-s001.zip › Figure S1 electropherogram.tif]

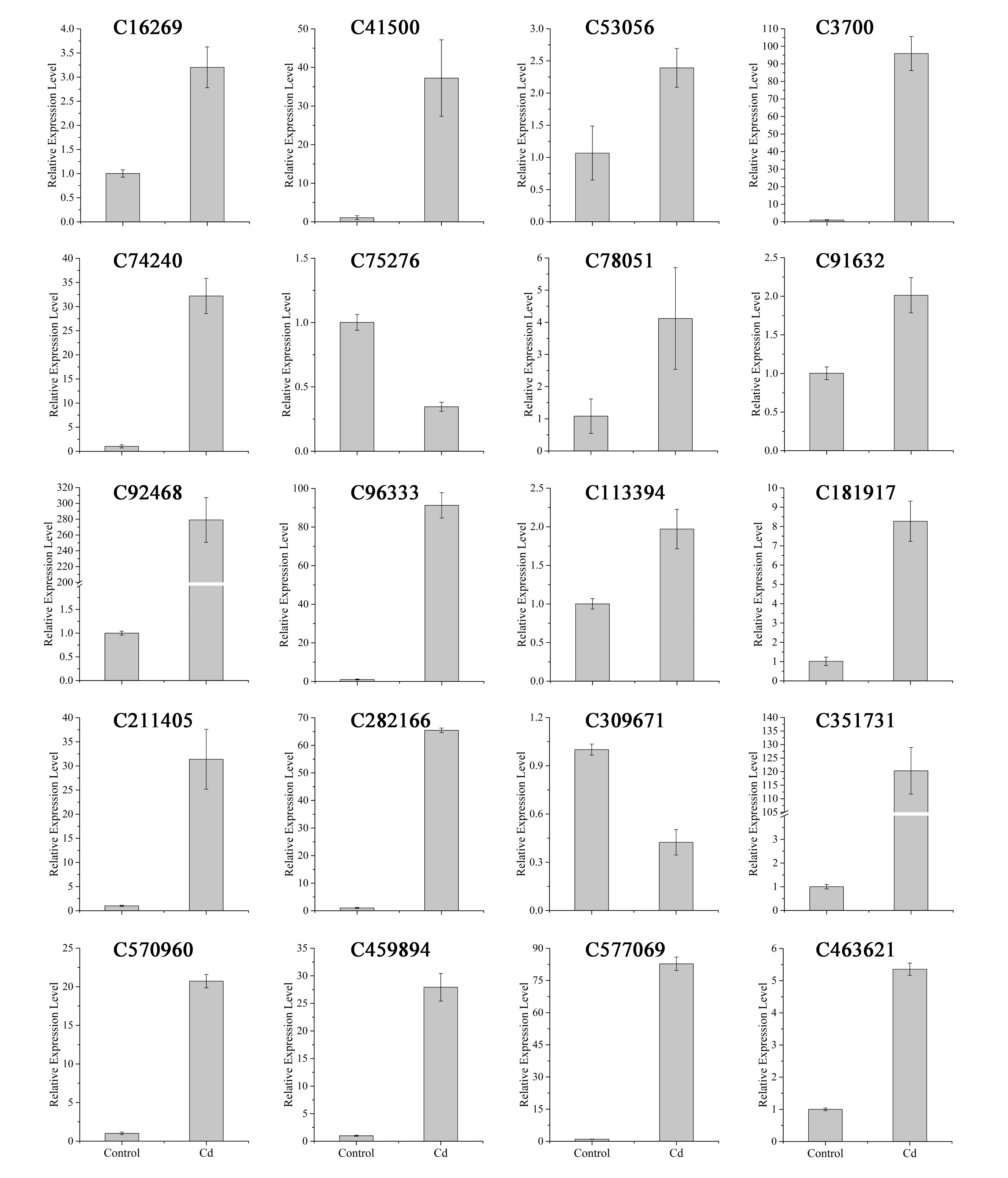

Supplement: Supplementary file 1 [file ijms-21-01067-s001.zip › Figure S2 Validation of DEGs with qRT-PCR.jpg]

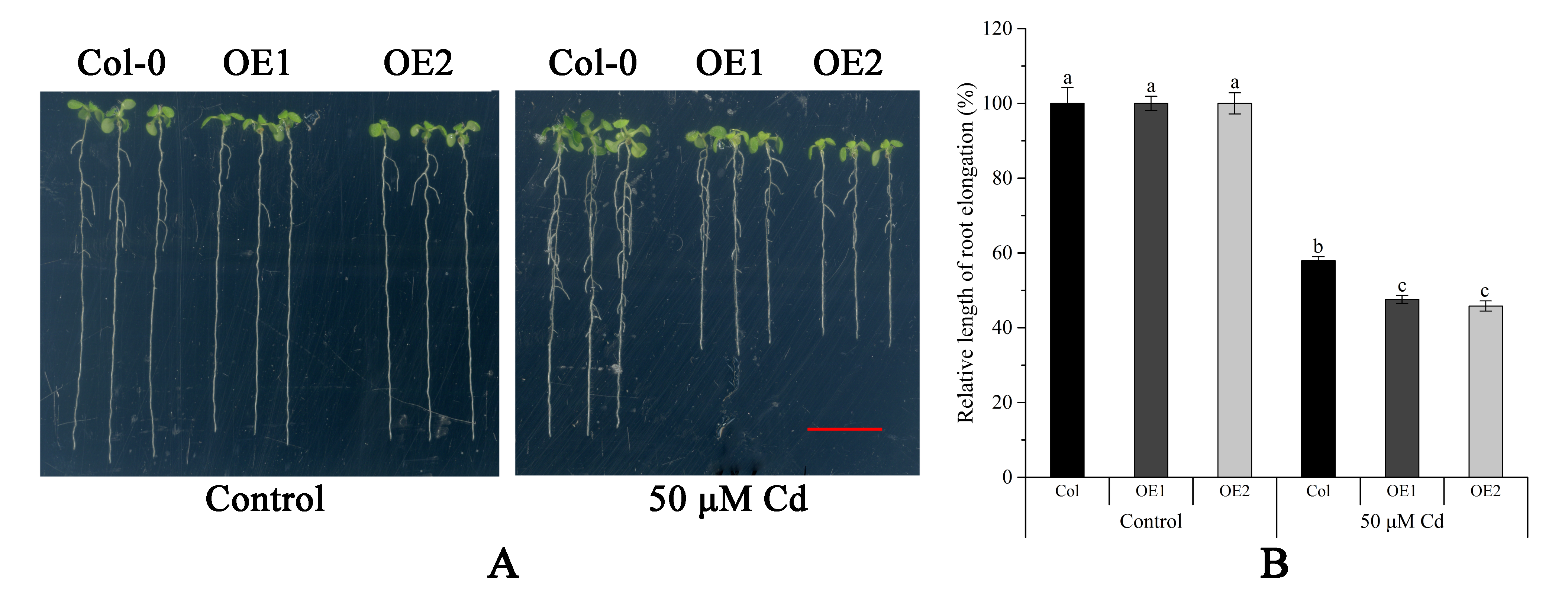

Supplement: Supplementary file 1 [file ijms-21-01067-s001.zip › Figure S3 The Cd tolerance experiment on plates of LmAUX1.tif]

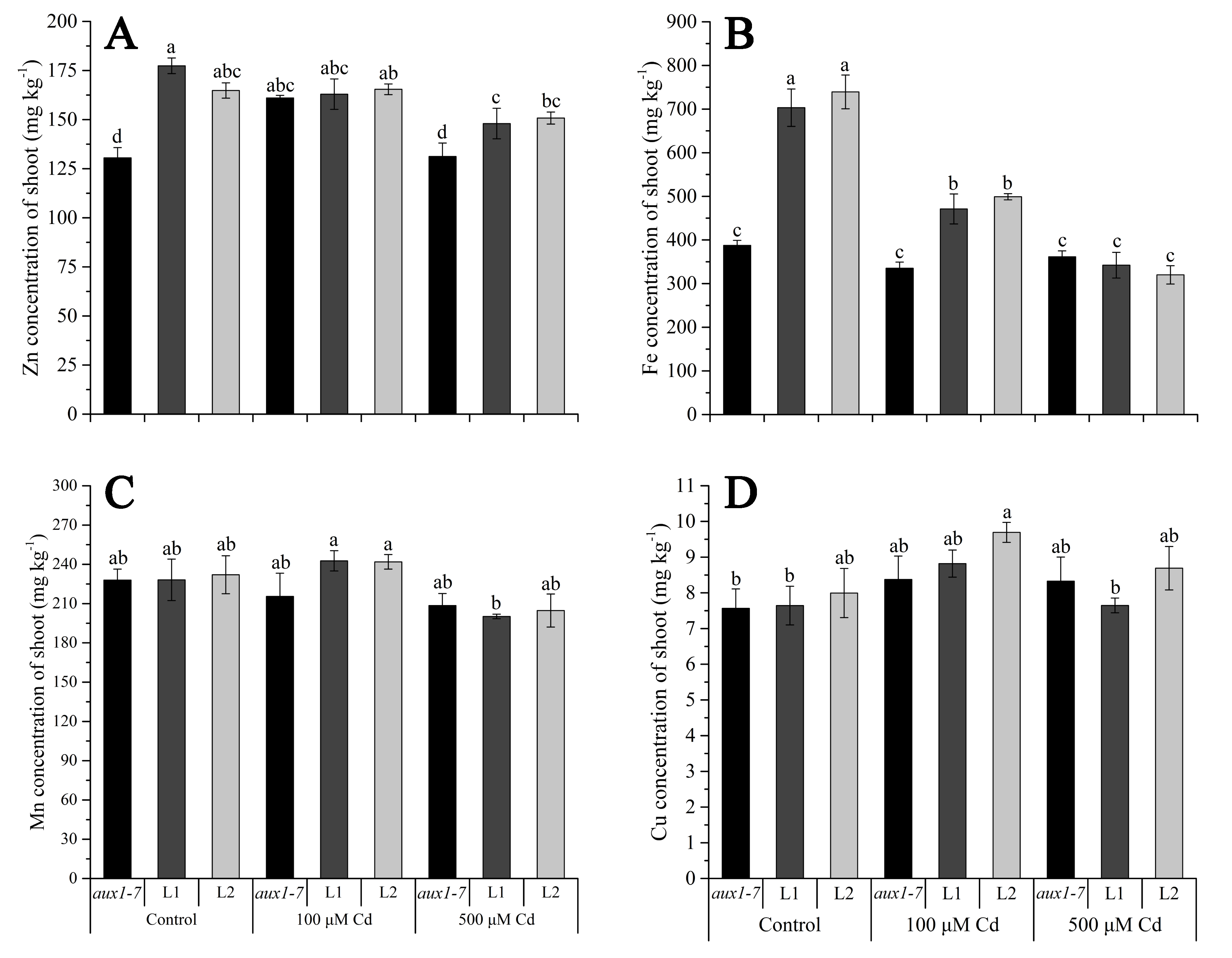

Supplement: Supplementary file 1 [file ijms-21-01067-s001.zip › Figure S5 Zn Fe Mn and Cu concentrations of aux1-7 shoot in soil experiment.tif.tif]

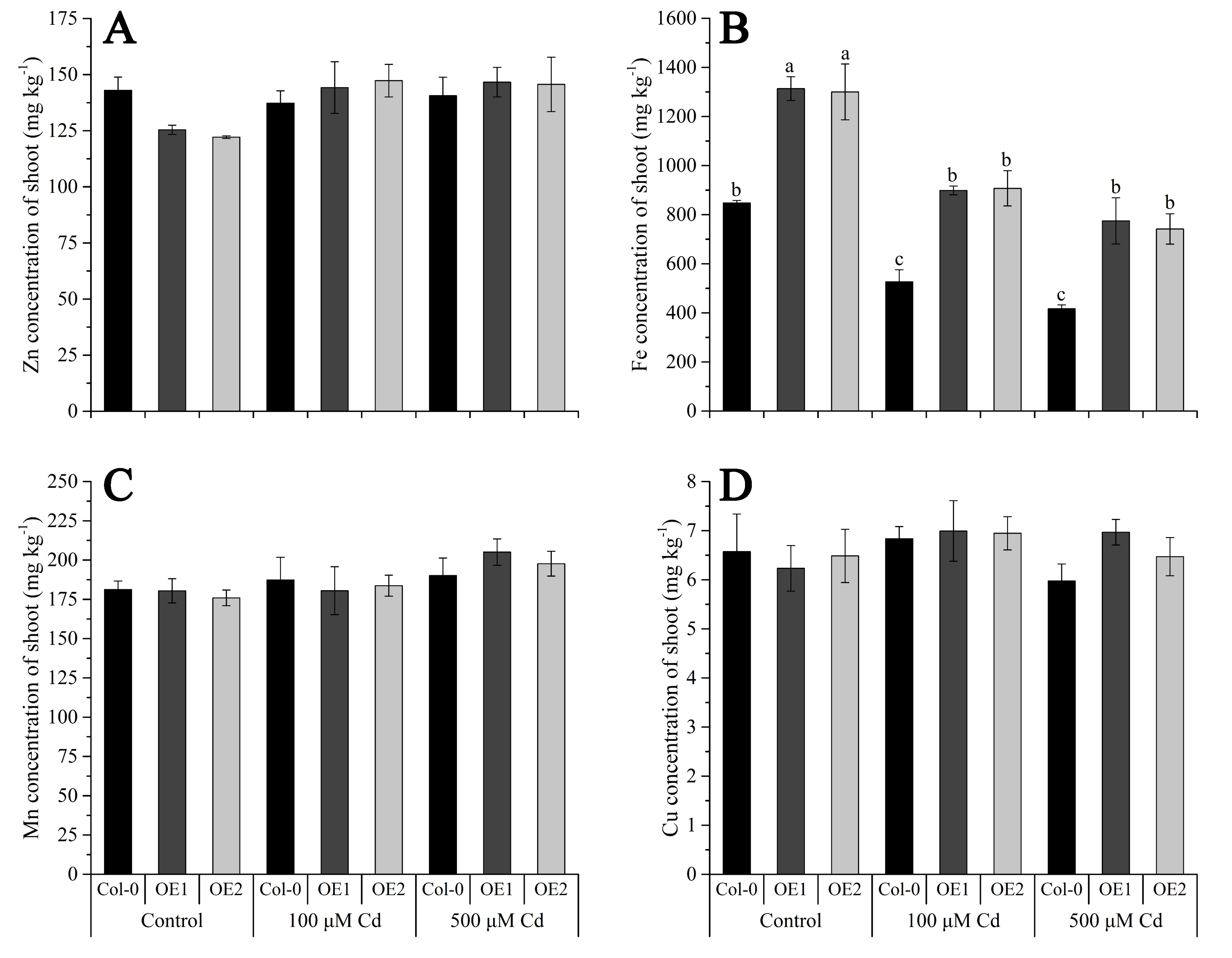

Supplement: Supplementary file 1 [file ijms-21-01067-s001.zip › Figure S6 Zn Fe Mn and Cu concentrations of col-0 shoot in soil experiment.tif]

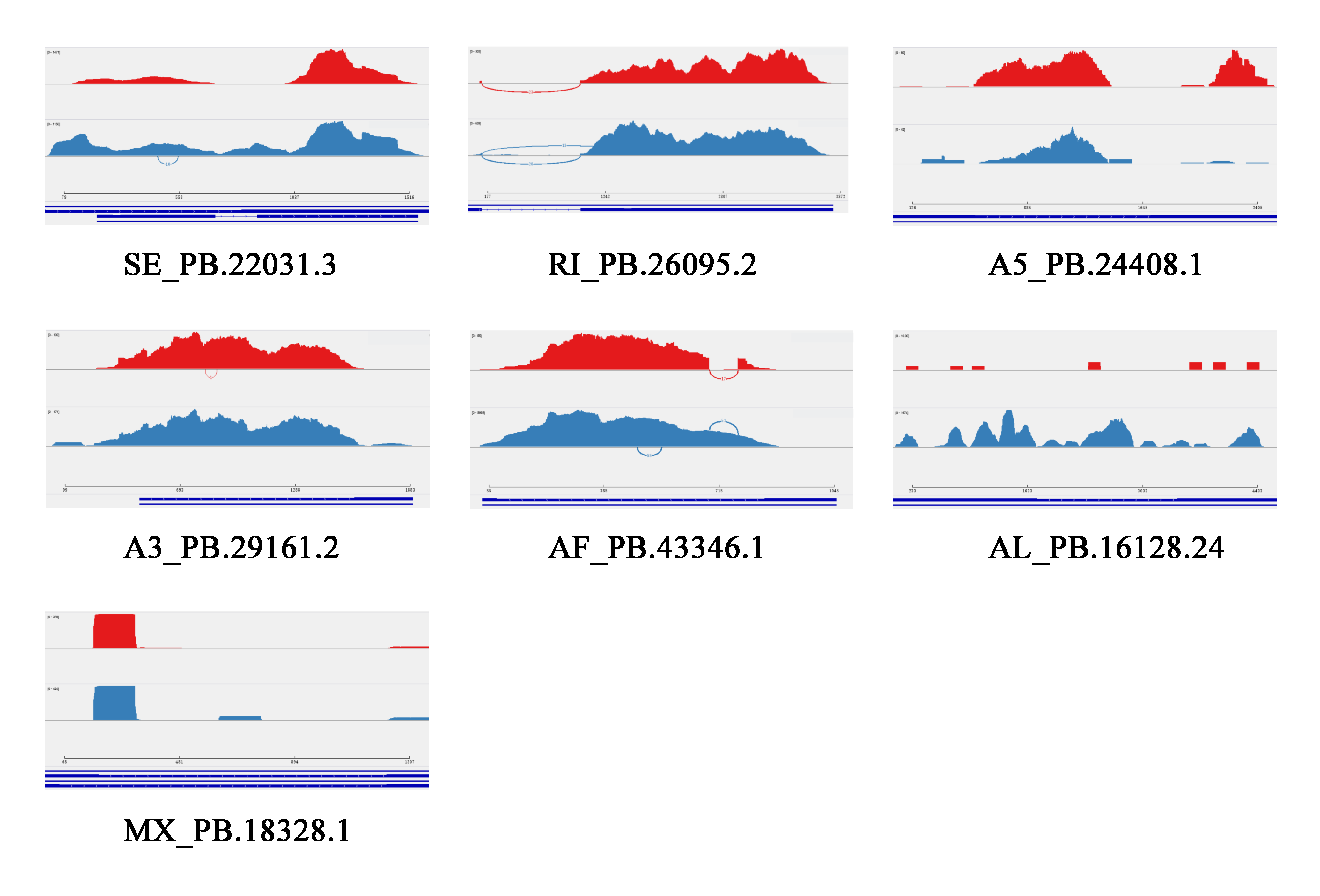

Supplement: Supplementary file 1 [file ijms-21-01067-s001.zip › Figure S7 Sashimi plots showing examples of the different alternative splicing (AS) events detected with PacBio ISO-seq.tif]

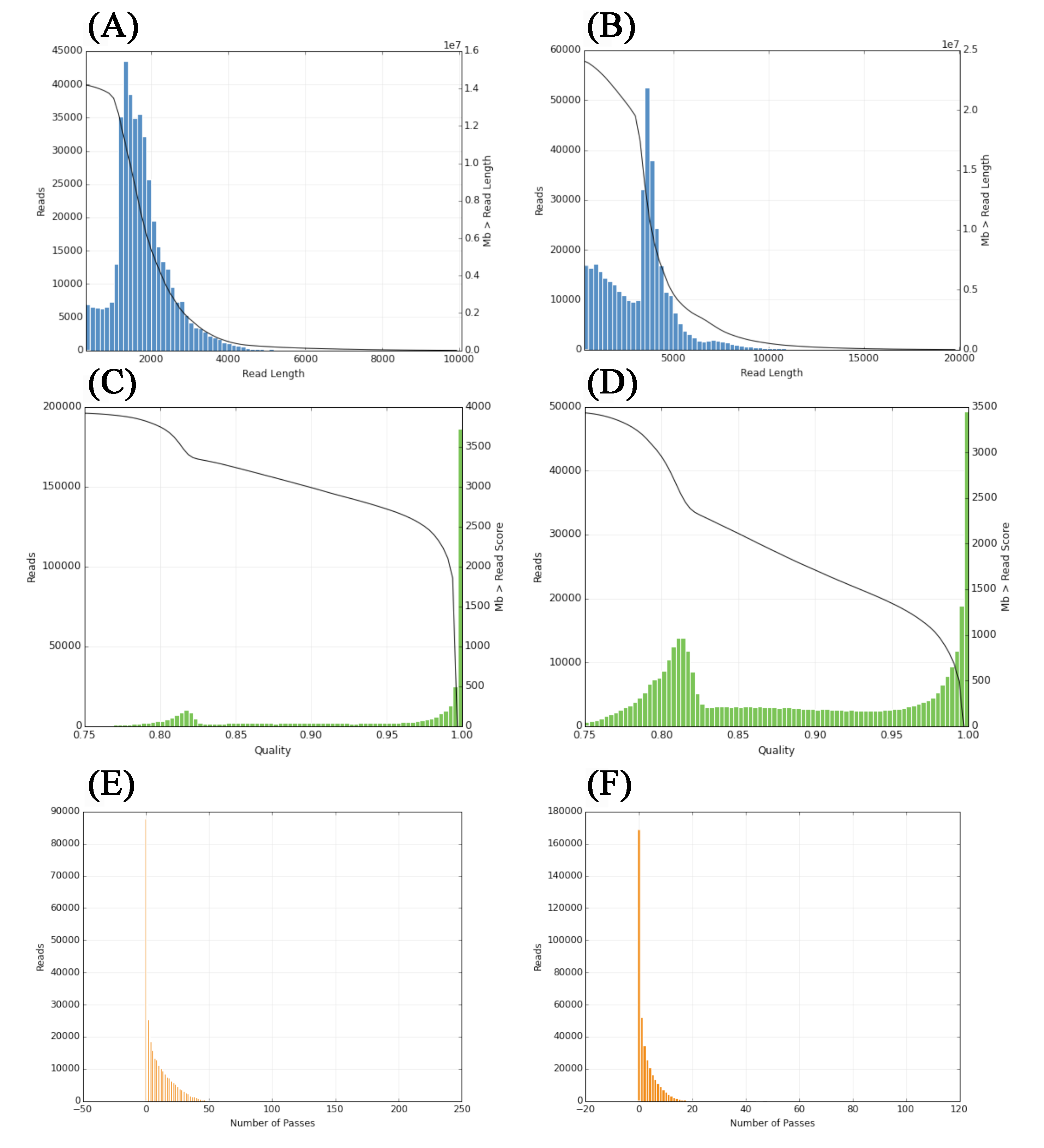

Supplement: Supplementary file 1 [file ijms-21-01067-s001.zip › Figure S8 Quality control of ROIs in individual libraries.tif]

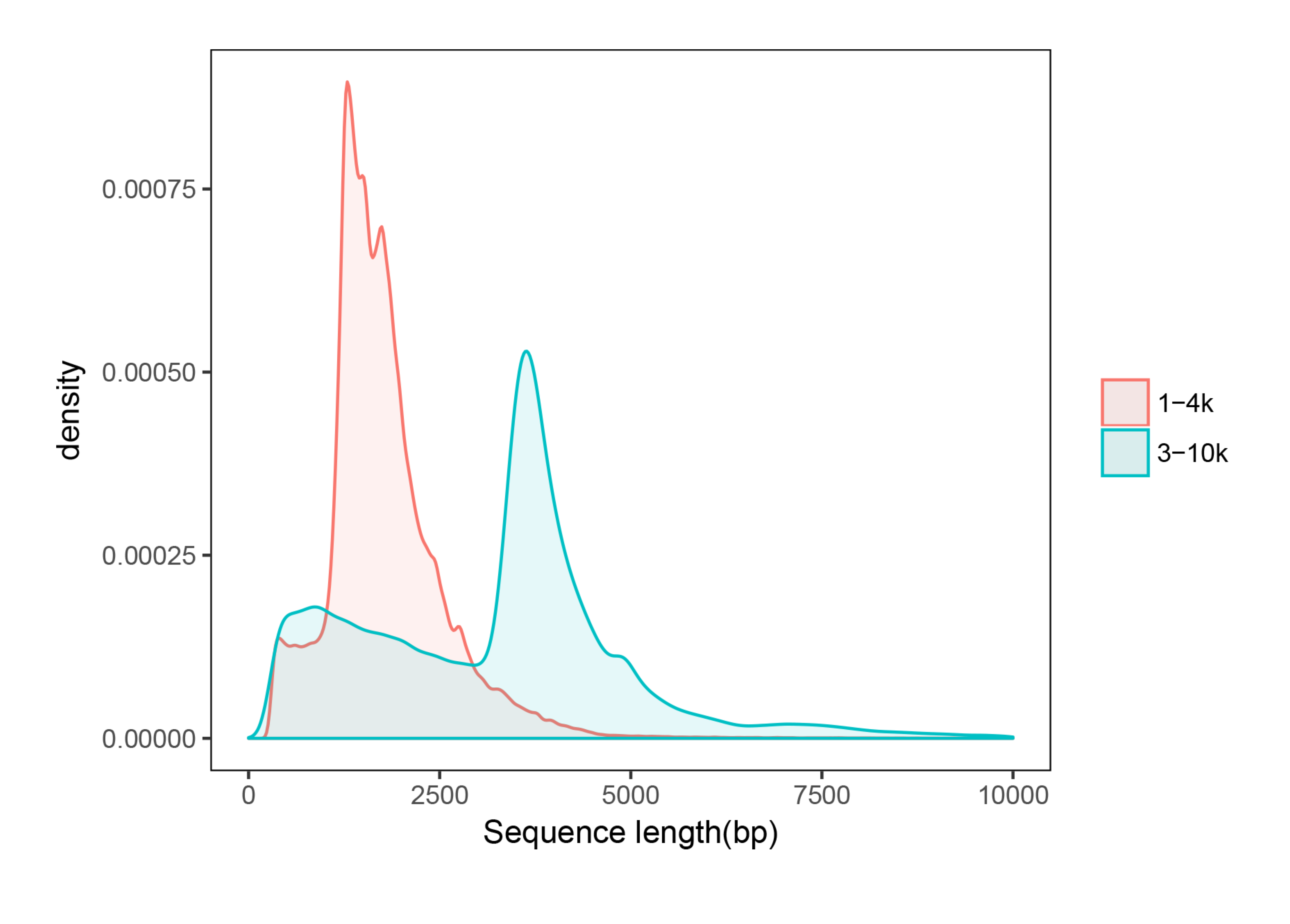

Supplement: Supplementary file 1 [file ijms-21-01067-s001.zip › Figure S9 Length density distribution of full-length non-chimeric (FLNC).tif]

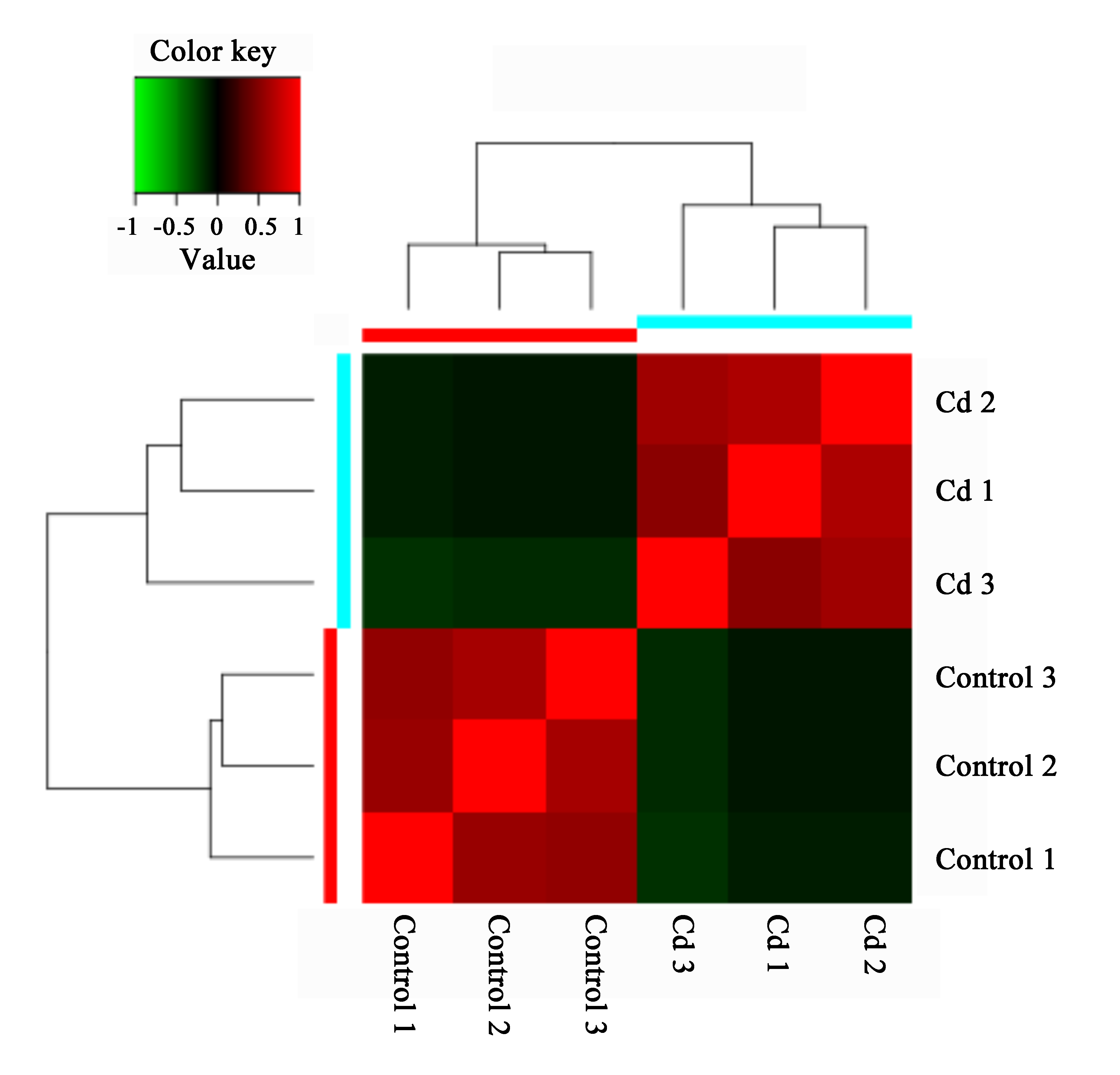

Supplement: Supplementary file 1 [file ijms-21-01067-s001.zip › Figure S10 Hierarchial cluster RNA-Seq samples of Control and Cd.tif]

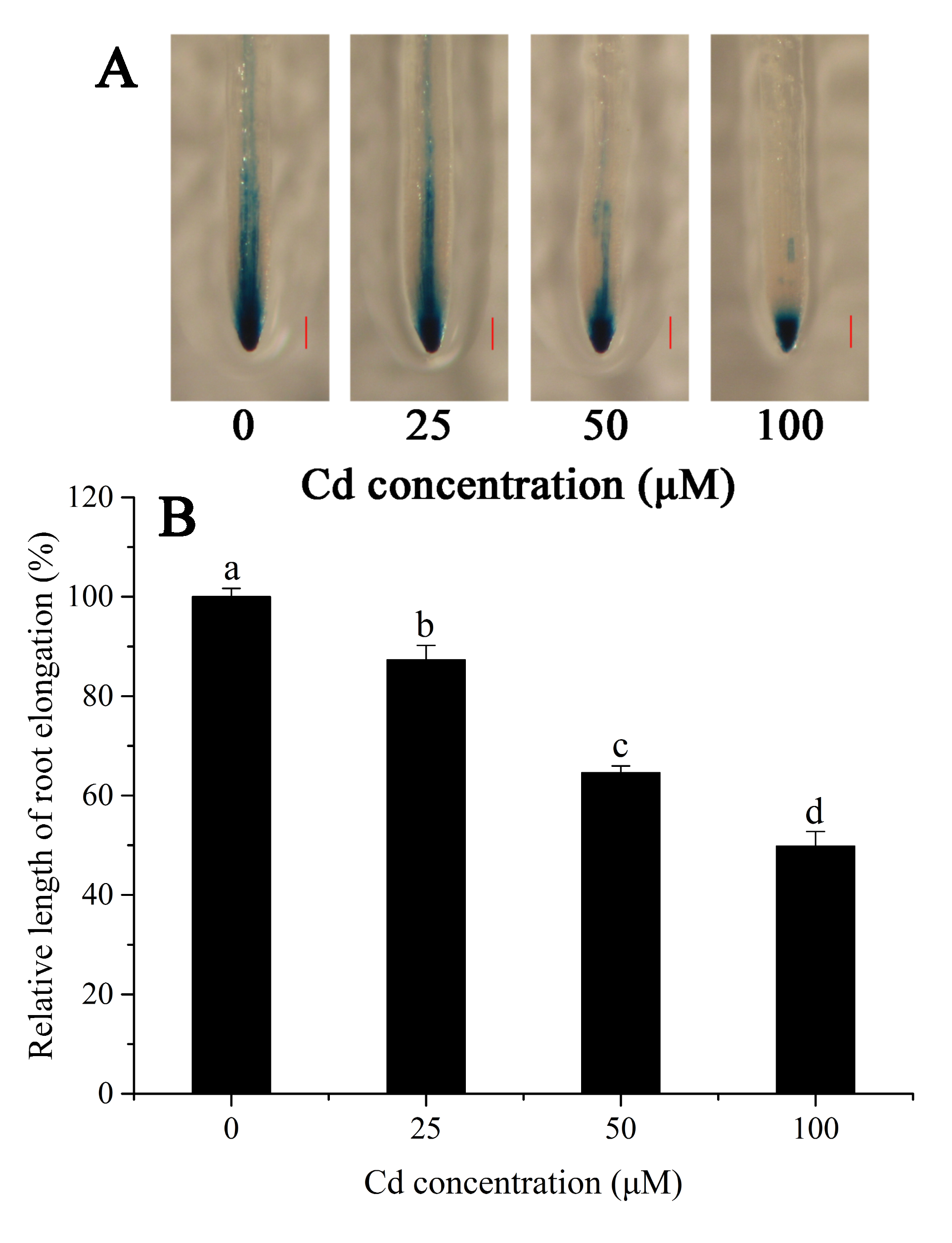

Supplement: Supplementary file 1 [file ijms-21-01067-s001.zip › Figure S11 Effects of Cd stress on the auxin level of Arabidopsis thaliana seedlings monitored by DR5GUS.tif]

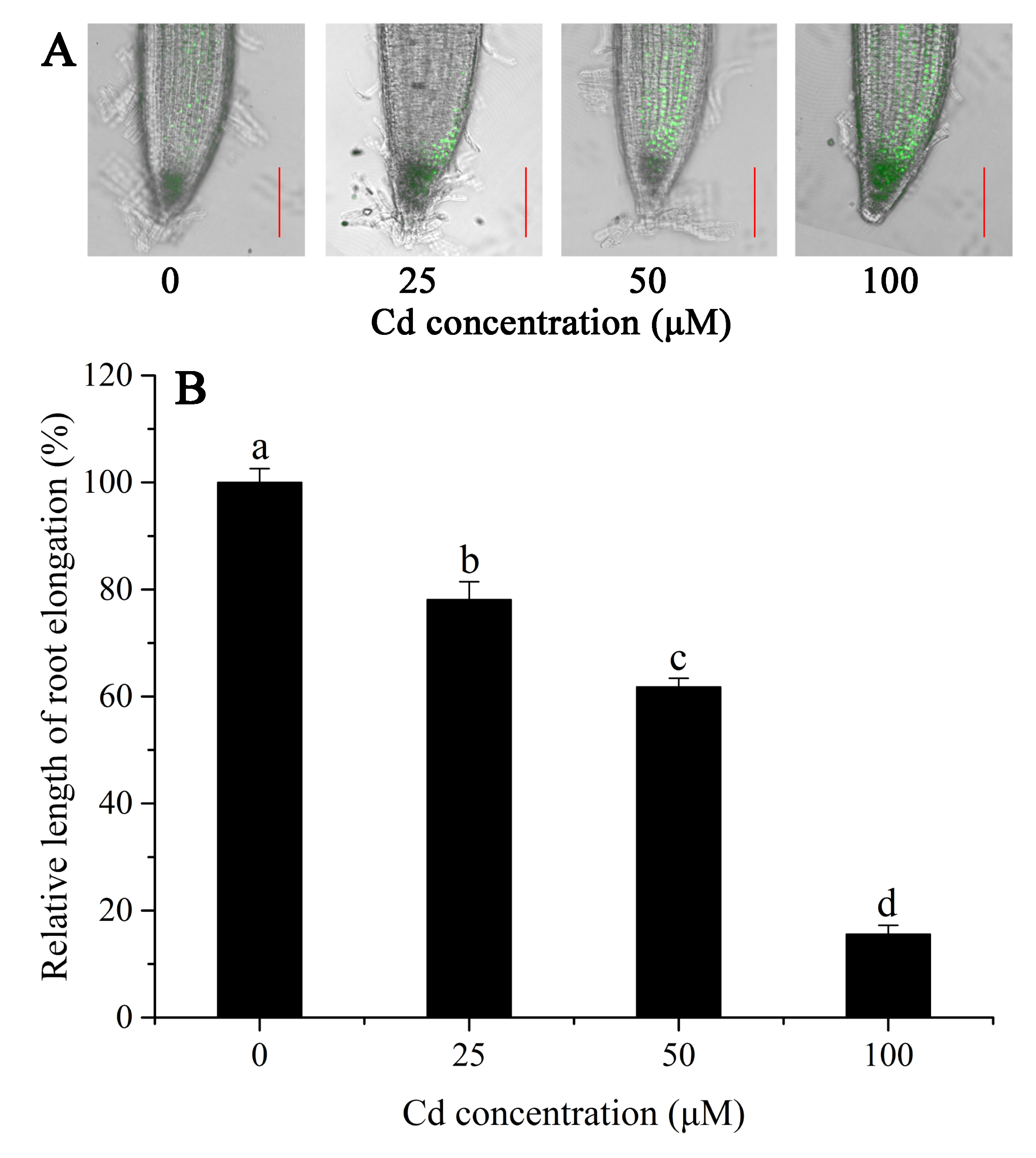

Supplement: Supplementary file 1 [file ijms-21-01067-s001.zip › Figure S12 Effects of Cd stress on the auxin level of Arabidopsis thaliana seedlings monitored with an auxin signaling sensor line, DII-VENUS.tif]

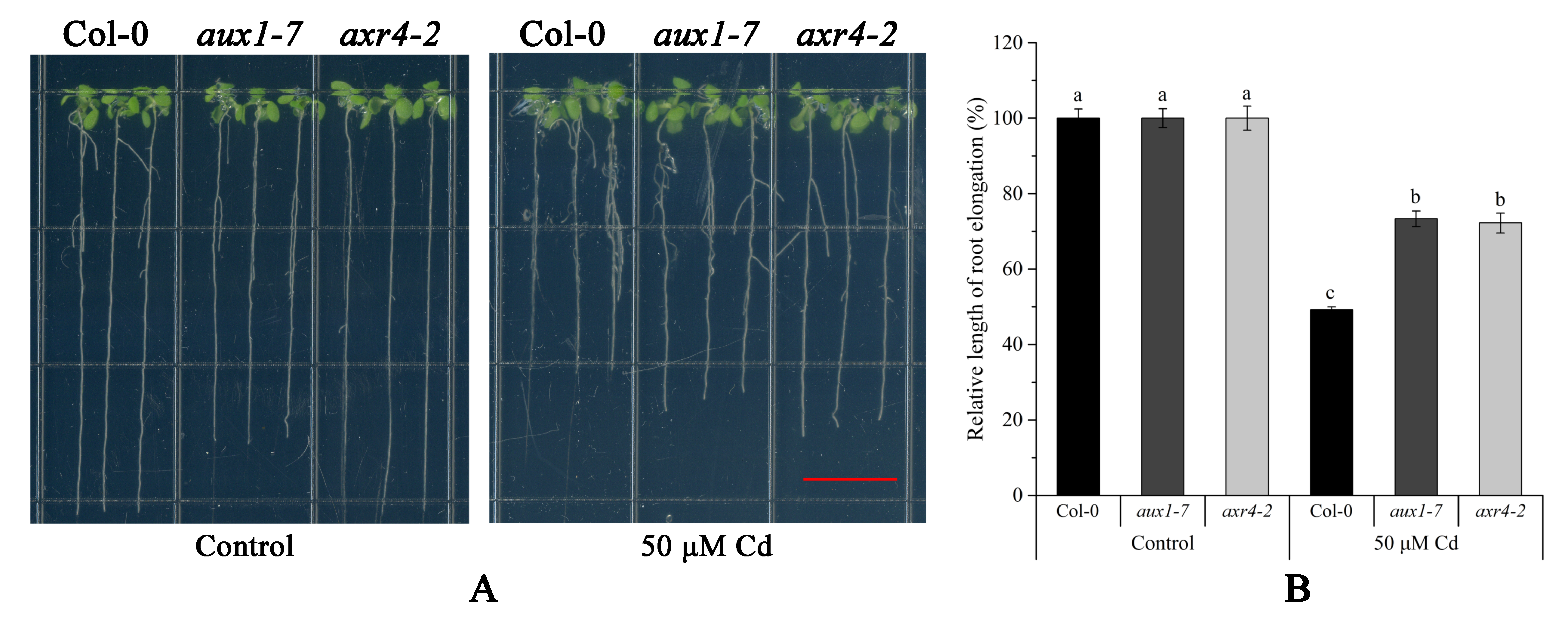

Supplement: Supplementary file 1 [file ijms-21-01067-s001.zip › Figure S13 Cd tolerance experiment on plates of auxin transporter mutant..tif]

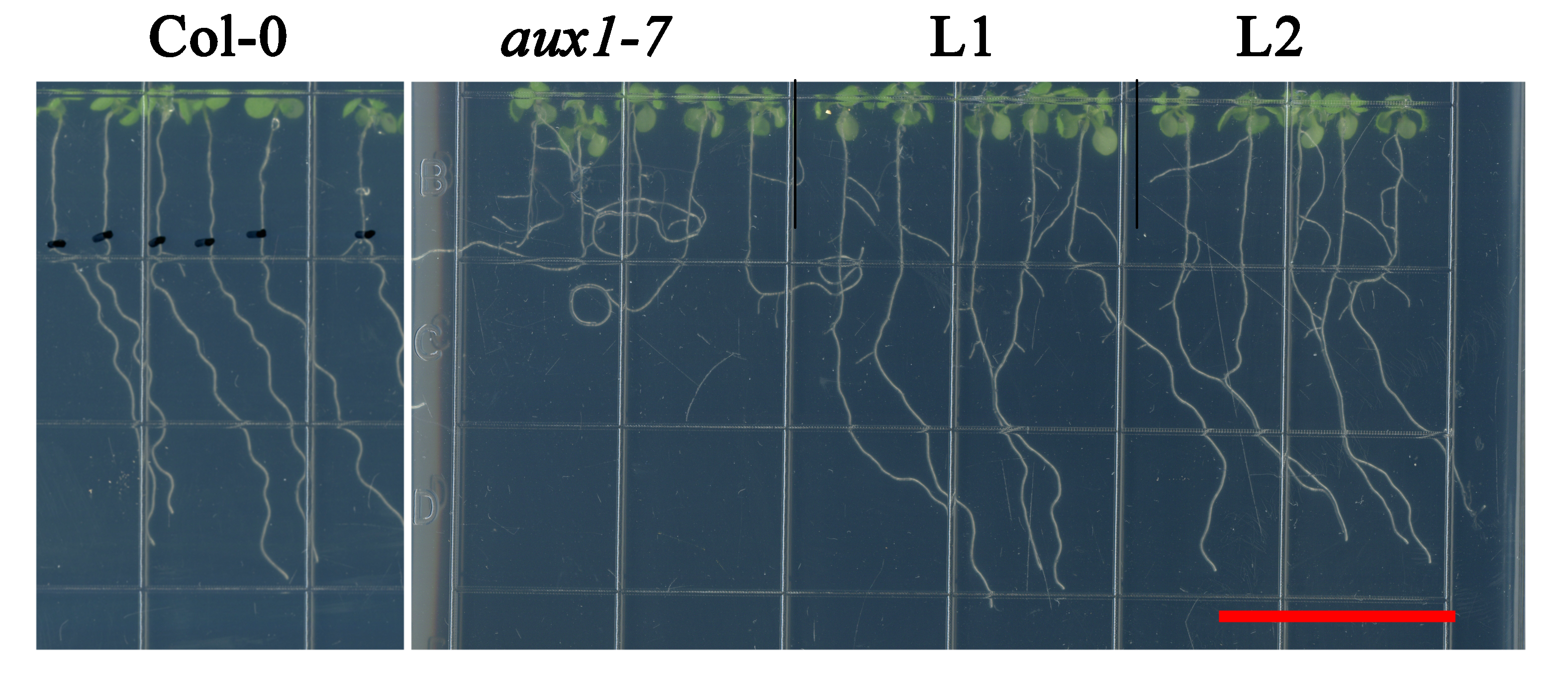

Supplement: Supplementary file 1 [file ijms-21-01067-s001.zip › Figure S14 Phenotype of 3-4 day Arabidopsis thaliana seedlings.tif]
