# Supplementary material for: Full-Length Transcriptome Assembly of Italian Ryegrass Root Integrated with RNA-Seq to Identify Genes in Response to Plant Cadmium Stress
Source: Int J Mol Sci. 2020 Feb 6;21(3):1067. doi: 10.3390/ijms21031067 (PMC7037684; doi:10.3390/ijms21031067)
Supplement: Supplementary file 1 [file ijms-21-01067-s001.zip › Supplementary Materials_clean.doc]

# Supplementary materials:

# Full-Length Transcriptome Assembly of Italian Ryegrass Root Integrated with RNA-Seq to Identify Genes in Response to Plant Cadmium Stress

Zhaoyang Hu, Yufei Zhang, Yue He, Qingqing Cao, Ting Zhang, Laiqing Lou*, and Qingsheng Cai

College of Life Sciences, Nanjing Agricultural University, Nanjing 210095, China; 2016216002@njau.edu.cn (Z.H); 2018116014@njau.edu.cn (Y.Z.); 2018116015@njau.edu.cn (Y.H.); 2016116018@njau.edu.cn (Q.C.); 2016116017@njau.edu.cn (T.Z.); loulq@njau.edu.cn (L.L.); qscai@njau.edu.cn (Q.C.)

*****	Correspondence: loulq@njau.edu.cn; Tel.: +86-25-84396408 (L.L.)

Received: date; Accepted: date; Published: date

**Supporting tables**

**Table S1**. Statistical non-chimeric reads information of PacBio ISO-seq ROI data

| Library | 1–4k | 3–10k |
| --- | --- | --- |
| Number of reads of insert | 415985 | 386341 |
| Number of 5’ reads | 307137 | 200048 |
| Number of 3’ reads | 347030 | 256046 |
| Number of poly(A) reads | 327086 | 209010 |
| Number of filtered short reads | 0 | 0 |
| Number of non-full-length reads | 128703 | 229999 |
| Number of full-length reads | 287282 | 156342 |
| Number of full-length non-chimeric reads | 270276 | 106080 |
| Full-length percentage (FL%) | 69.06% | 40.47% |
| Average full-length non-chimeric read length | 1711 | 3540 |

**Table S2**. Information on polished consensus isoforms

| Library | Number of consensus isoforms | Average consensus isoforms read lengths | Number of polished high-quality isoforms | Number of polished low-quality isoforms |
| --- | --- | --- | --- | --- |
| 1-4k | 118679 | 1756 | 21433 | 97050 |
| 3-10k | 62515 | 3653 | 8307 | 54200 |

**Table S3**. Summary of the RNA-Seq raw data

| Sample Name | Clean Reads | Clean Bases | Clean N | Clean GC | Clean Q20 | Clean Q30 |
| --- | --- | --- | --- | --- | --- | --- |
| Control-1 | 48 302 746 | 14 490 823 800 | 0.01;0.01 | 57.43;57.30 | 98.43;94.24 | 95.30;87.18 |
| Control-2 | 48 703 516 | 14 611 054 800 | 0.01;0.01 | 57.59;57.53 | 98.41;94.15 | 95.27;87.02 |
| Control-3 | 39 3404 73 | 11 802 141 900 | 0.01;0.01 | 57.44;57.36 | 98.44;94.20 | 95.31;87.12 |
| Cd-1 | 51 082 858 | 15 324 857 400 | 0.01;0.01 | 57.26;57.13 | 98.40;94.08 | 95.29;86.91 |
| Cd-2 | 44 120 951 | 132 362 85 300 | 0.01;0.01 | 58.09;57.96 | 98.40;94.22 | 95.24;87.16 |
| Cd-3 | 44 917 284 | 134 751 85 200 | 0.00;0.01 | 57.98;57.87 | 98.38;94.36 | 95.20;87.53 |

**Table S4.** Information of raw data

| Library | Polymerase Read Bases | Polymerase Reads | Polymerase Read N50 | Polymerase Read Length |
| --- | --- | --- | --- | --- |
| 1-4k | 8.12GB | 438249 | 35250 | 18537 |
| 3-10k | 5.52GB | 421542 | 27250 | 13085 |

Polymerase read bases: raw databases (filtered);

Polymerase reads: numbers of polymerase reads (filtered);

Polymerase read N50: length of polymerase read N50 (filtered);

Polymerase read length: average length of polymerase read (filtered).

**Table S5.** Information of ROI

| Library | Reads of insert | Read bases of insert | Mean read length of insert | Mean read quality of insert | Mean number of passes |
| --- | --- | --- | --- | --- | --- |
| 1–4k | 415985 | 737360471 | 1772 | 0.945 | 10 |
| 3–10k | 386341 | 1257989128 | 3256 | 0.8920 | 3 |

Reads of insert: number of ROI;

Read bases of insert: total base number of ROI;

Mean read length of insert: mean length of ROI;

Mean read quality of insert: mean quality of ROI;

Mean number of passes: mean complete reads of ROI.

**Table S6.** Primers used in this study

| Name | 5’ → 3’ sequence | Description | Purpose |
| --- | --- | --- | --- |
| C16269-F | CGCGTATGACTATGCACCCT | Multiflorum_3-10k_c16269_f1p18_838 | qRT-PCR |
| C16269-R | CGTCGCCTTTGTTGAGCATC |  |  |
| C53056-F | CCGAACCGAACAGTCCAGAA | Multiflorum_3-10k_c53056_f1p19_913 | qRT-PCR |
| C53056-R | TGTCCACGACGCAAAAGTCT |  |  |
| C75276-F | TGATCTTCGCGCTCGTCTAC | Multiflorum_3-10k_c75276_f1p284_1251 | qRT-PCR |
| C75276-R | GTACAGACCCTGCTGGAACC |  |  |
| C309671-F | GTGTTCCAGGACACCGACAT | Multiflorum_1-4k_c309671_f2p10_1749 | qRT-PCR |
| C309671-R | GCGTTGAAGTTCCCCAAACC |  |  |
| C92468-F | CCAAGGAGGAGGCCAAGAAG | Multiflorum_3-10k_c92468_f5p222_784 | qRT-PCR |
| C92468-R | CAGACACTAGACCCCGAGGA |  |  |
| C459894-F | AAGGAGCTGTGCAAGAGCAT | Multiflorum_1-4k_c459894_f3p21_1884 | qRT-PCR |
| C459894-R | ACATCGAGCAGAAGCAGGTC |  |  |
| C282166-F | CGAGGATGGCAAGATCGACA | Multiflorum_1-4k_c282166_f4p8_1067 | qRT-PCR |
| C282166-R | ATCCTTGAGGGTGGCATTGG |  |  |
| C78051-F | AGAACGCGCTGGAGAACTAC | Multiflorum_1-4k_c78051_f30p35_2279 | qRT-PCR |
| C78051-R | GTCGATCGCGTCCTCAATCT |  |  |
| C41500-F | TCGATATCAAGTTCCCGGCG | Multiflorum_1-4k_c41500_f5p10_1435 | qRT-PCR |
| C41500-R | CCAACACACACACGAGAGGA |  |  |
| C181917-F | ACGACACCGACAACCTCATC | Multiflorum_1-4k_c181917_f1p13_1233 | qRT-PCR |
| C181917-R | CGTGAGGAGGGAGAGGAAGA |  |  |
| C463621-F | CATCTCTCGTGAGACGCTCC | Multiflorum_1-4k_c463621_f5p9_2379 | qRT-PCR |
| C463621-R | TTGATGCCGAGCTTCAGGTT |  |  |
| C211405-F | GGGCTCGAGGTGAACAAGAA | Multiflorum_1-4k_c211405_f7p10_1286 | qRT-PCR |
| C211405-R | AATCACAGAGCCTCAAGCCC |  |  |
| C91632-F | TTTTCCTAGCGAACCCCGAC | Multiflorum_1-4k_c91632_f1p58_1289 | qRT-PCR |
| C91632-R | CCGACGACGGGATCAGAAAT |  |  |
| C570960-F | TGGTGGAGACGAACTGAAGC | Multiflorum_1-4k_c570960_f1p1_844 | qRT-PCR |
| C570960-R | TACTCGTAGCTCAGGCCCTT |  |  |
| C3700-F | GACAACGCTGGGTATGTGGA | Multiflorum_3-10k_c73700_f1p11_986 | qRT-PCR |
| C3700-R | GGTCTTGACGCCCTTCATCA |  |  |
| C351731-F | TCGACAAGGTGGAGAAGCAC | Multiflorum_1-4k_c351731_f15p21_1764 | qRT-PCR |
| C351731-R | CAGGCCTTGAGACCCTTGAG |  |  |
| C74240-F | AGGCTCAAGCAACATCGGAA | Multiflorum_3-10k_c74240_f1p2_798 | qRT-PCR |
| C74240-R | ATGTCTGCAGCTTGGAGGTC |  |  |
| C113394-F | TGACATGGCCGGAGATGATG | Multiflorum_3-10k_c113394_f2p170_643 | qRT-PCR |
| C113394-R | ATGATTGGCACCTGGGACTG |  |  |
| C577069-F | GTTCAAGCGCAAGCACAAGA | Multiflorum_3-10k_c113394_f2p170_643 | qRT-PCR |
| C577069-R | CGAGTCGATCTCGATGGTGG |  |  |
| C96333-F | AAGGCGAATGGGAGATGGTG | Multiflorum_3-10k_c96333_f1p10_914 | qRT-PCR |
| C96333-R | TCTCCTCCTGTCTCCTCTGC |  |  |
| eEF1A-F | GATGATTCCCACCAAGC | reference gene for *Lolium multiflorum* L. | qRT-PCR |
| eEF1A-R | TAGTAGCAGACAACCACCAG |  |  |
| ACTIN2-F | GCTCCTCTTAACCCAAAGGC | reference gene for *Arabidopsis thaliana* | qRT-PCR |
| ACTIN2-R | CACACCATCACCAGAATCCAGC |  |  |
| AUX1-F | aagtccggagctagctctagaATGGTTCCCGGCGATCAC | Multiflorum_1-4k_c11285_f6p6_2149 | Vector construction |
| AUX1-R | gcccttgctcaccatggatccCTAGTGGTGCGGCGGCGC |  |  |

**Supporting figures**

**Figure S1.** Gel electrophoresis of total cDNA. The first lane was the 10 KB DNA marker and the sample was diffuse in 7RS466 lane.

**Figure S2.** Validation of DEGs with qRT-PCR. Data are means ± SE (n=4).

**Figure S3.** The Cd tolerance experiment on plates of *LmAUX1*. *LmAUX1* was transformed into the *Arabidopsis thaliana* wild type (Col-0) with the *CaMV35S* promoter, and two lines (OE1 and OE2) were used for the experiment. (A) Phenotype of plants grown in 1/2 MS medium with or without 50 μM Cd for 4–5 d. Scale bar =1 cm. (B) Relative length of root elongation. Data are means ± SE (n ≥ 15); different letters above the bars were significantly different at *P* < 0.05 (Duncan’s test).

**Figure S4.** Cd translocated experiment in soil of *LmAUX1*. *LmAUX1* was transformed into *Arabidopsis thaliana* wild type (Col-0) with the *CaMV35S* promoter, and two lines (OE1 and OE2) were used for the experiment. Plants grown in soils irrigated with 0, 100 or 500 μM Cd solution for 3–4 weeks. Data are means ± SE (n = 3); different letters above the bars are significantly different at *P* < 0.05 (Duncan’s test).

**Figure S5.** Zn (A), Fe (B), Mn (C), and Cu (D) concentrations of *Arabidopsis thaliana* shoot in *aux1-7* mutant soil experiment. *LmAUX1* was transformed into the *Arabidopsis thaliana aux1-7* mutant with the *CaMV35S* promoter, and two lines (L1 and L2) were used for the experiment. Data are means ± SE (n = 3); different letters above the bars are significantly different at *P* < 0.05 (Duncan’s test).

**Figure S6.** Zn (A), Fe (B), Mn (C), and Cu (D) concentrations of *Arabidopsis thaliana* shoot in Col-0 soil experiment. *LmAUX1* was transformed into Arabidopsis thaliana wild type (Col-0) with the CaMV35S promoter, and two lines (OE1 and OE2) were used for the experiment. Data are means ± SE (n = 3); different letters above the bars are significantly different at *P* < 0.05 (Duncan’s test).

**Figure S7.** Sashimi plots showing examples of the different alternative splicing (AS) events detected with PacBio ISO-seq. Peaks in red (Control) and blue (Cd) represent the coverage of RNA-seq short reads and curved lines with numbers represent splicing junctions supported by that number of short reads. Blue lines below the peaks indicate different isoforms, blocks in blue represent exons and lines in-between represent introns.

**Figure S8.** Quality control of ROIs in individual libraries. Length distribution of ROIs in 1-4k (A) and 3-10k (B) libraries, the curves of the right y-axis indicate the base numbers of all sequences which the length less than the corresponding value of x-axis. Quality distribution of ROIs in 1-4k (C) and 3-10k (D) libraries, the curves of right y axis represent the base numbers of all sequences which the quality less than the corresponding value of x axis. Number of Full passes distribution of ROIs in 1-4k (E) and 3-10k (F) libraries.

**Figure S9.** Length density distribution of full-length non-chimeric (FLNC) reads in 1–4k (read curves) and 3–10k (blue curves) libraries.

**Figure S10.** Hierarchical cluster RNA-seq samples based on the fragments per kilobase of exon per million fragments mapped (FPKM) values.

**Figure S11.** Effects of Cd stress on the auxin level of *Arabidopsis thaliana* seedlings monitored by *DR5::GUS*. (A) GUS activity in primary root apices in 5-day-old *DR5::GUS* seedlings treated with 0, 25, 50, 100 μM Cd for 3-4 days on 1/2 MS plates. Scale bars = 100 μm. (B) Relative length of root elongation of *Arabidopsis thaliana* *DR5::GUS* seedlings grown on 1/2 MS plates supplied with 0, 25, 50, 100 μM Cd for 3-4 days on 1/2 MS plates. Data are means ± SE (n ≥ 15), different letters above the bars were significantly different at *P* < 0.05 (Duncan’s test).

**Figure. S12.** Effects of Cd stress on the auxin level of *Arabidopsis thaliana* seedlings monitored with an auxin signaling sensor line, *DII-VENUS*. (A) auxin signaling level in primary root apices in 5-day-old *Arabidopsis thaliana* *DII-VENUS* seedlings treated with 0, 25, 50, 100 μM Cd for 3-4 days on 1/2 MS plates. Scale bars = 100 μm. (B) Relative length of root elongation of *Arabidopsis thaliana* *DII-VENUS* seedlings grown on 1/2 MS plates supplied with 0, 25, 50, 100 μM Cd for 3-4 days on 1/2 MS plates. Data are means ± SE (n ≥ 15), different letters above the bars were significantly different at *P* < 0.05 (Duncan’s test).

**Figure S13.** Cd tolerance experiment on plates of auxin transporter mutant. (A) Phenotype of *Arabidopsis thaliana* seedlings (Col-0 wild type and two mutants *aux1*-7, *axr4*-2) grown on 1/2 MS medium with or without 50 μM Cd for 4-5 d, scale bar = 1 cm. (B) Relative length of root elongation, data are means ± SE (n ≥ 15), different letters above the bars were significantly different at *P* < 0.05 (Duncan’s test).

**Figure S14.** Phenotype of 3-4 day *Arabidopsis thaliana* seedlings (Col-0 wild type, *aux1-7* mutant and two transformed *lmAUX1* into *aux1-7* lines) grown on 1/2 MS medium for 4-5 d, scale bar = 2 cm.
